# Discovery of Quercetin as a Potential Entry Inhibitor of Nipah Virus: A Path Toward Antiviral Therapy

**DOI:** 10.3390/ijms27188026

**Published:** 2026-09-09

**Authors:** Mohammad Mamun Alam, Md. Mohibur Rahman, Khalid Hasan Raj, Abdul Hadi Nahid, Poulomi Saha, Abir Hossain, Moushimi Amaya, Eric D. Laing, Syed Moinuddin Satter, Christopher C. Broder, Mohammad Enayet Hossain, Mohammed Ziaur Rahman

**Affiliations:** 1 Infectious Diseases Division, International Centre for Diarrhoeal Disease Research (icddr,b), Dhaka 1212, Bangladesh; mamun.alam@icddrb.org (M.M.A.); mohibur.rahman@icddrb.org (M.M.R.); khalid.raj@du.ac.bd (K.H.R.); abdul.nahid@icddrb.org (A.H.N.); poulomi.saha@icddrb.org (P.S.); abir29.me@gmail.com (A.H.); dr.satter@icddrb.org (S.M.S.); mzrahman@icddrb.org (M.Z.R.); 2 Department of Microbiology and Immunology, Uniformed Services University, Bethesda, MD 20814, USA; moushimi.amaya@usuhs.edu (M.A.); eric.laing@usuhs.edu (E.D.L.); christopher.broder@usuhs.edu (C.C.B.)

**Keywords:** Nipah virus, G glycoprotein, quercetin, virtual screening, molecular dynamics simulation, small-molecule inhibitors

## Abstract

Nipah virus (NiV) is a zoonotic virus that causes severe encephalitis and respiratory disease with a mortality rate often exceeding 70%. Currently, there are no licensed vaccines or therapeutics for NiV disease. This study aimed to identify and experimentally evaluate small-molecule inhibitors targeting the NiV-G (attachment) glycoprotein using integrated in silico and in vitro approaches. A high-throughput virtual screening of >215,000 compounds was conducted against the NiV-G glycoprotein, using docking and molecular dynamics (MD) simulations. The next potential candidates were evaluated using an established, BSL-2-compatible recombinant Cedar virus (rCedV)-based green fluorescent protein (GFP) reporter virus expressing the NiV-F (fusion) and G glycoproteins (rCedV-NiV-B-GFP). During MD simulations, quercetin demonstrated the most stable binding, maintaining a consistent RMSD (3.25 ± 0.3 Å). In vitro testing showed dose-dependent inhibition of rCedV-NiV-B-GFP infection. Although quercetin alone was less potent (IC_50_ = 24 µM; 95% CI: 7.6–93.6 µM) than a reference NiV-neutralizing monoclonal antibody mAb-7B7 (IC_50_ = 0.027 μg/mL; approximately 0.00018 μM; 95% CI: 0.0108 to 0.0735), combination with ascorbic acid enhanced neutralization (IC_50_ = 4.4 µM; 95% CI: 2.9–6.8 µM). Quercetin demonstrated a high Selectivity Index of >20.8. The study identifies quercetin as a promising small-molecule inhibitor of NiV cellular infection, potentially through a stable interaction with NiV-G at the ephrin-B2/B3 binding interface identified computationally. These findings highlight the importance of combining computational screening with BSL-2 cell-based bioassays to accelerate NiV countermeasure discovery.

## 1. Introduction

Intensified interactions between human and animal health have increased the frequency of viral pathogen emergence and re-emergence [1,2]. The spillover of zoonotic viruses into human populations can lead to the emergence of novel strains with pandemic potential, posing a direct risk to global health security [3]. Nipah virus (NiV) is a zoonotic virus in the genus *Henipavirus* (family *Paramyxoviridae*) [4]. NiV is considered a priority pathogen with pandemic potential due to its high mortality rate, ability to transmit human-to-human, recurrent outbreaks, and lack of any approved countermeasures for use in people [4,5]. NiV was first identified as the causative agent of an outbreak of severe encephalitis among pig farmers in Peninsular Malaysia in 1998–1999, and subsequently shown to be genetically closely related to Hendra virus (HeV), which had been discovered just a few years prior in Brisbane, Australia [6]. Since then, near annual emergence of NiV follows a seasonal pattern in Bangladesh and India during the winter [7,8]. The natural reservoir of NiV is fruit bats in the *Pteropodidae* family. Primary transmission of NiV in Bangladesh is through fruit bat excreta that contaminates date palm juice subsequently consumed by humans, seeding outbreaks [7].

Infection with NiV has serious clinical and public health consequences. Case fatality rates (CFRs) have previously varied from 40% to 100% across reported outbreaks [9]. In recent years, NiV outbreaks in Bangladesh and India have led to cases and deaths surpassing the previous decade in total, with a peak CFR of 100% in 2024 [7,10,11]. NiV disease presents as a rapidly developing encephalitis that can be followed by serious respiratory problems [7,12,13]. NiV also poses considerable risk because of limited but potential human-to-human transmission, primarily via direct contact with respiratory secretions or fluids from infected individuals. Human-to-human NiV transmission has been a source of infection and outbreaks in community and healthcare settings [14,15].

NiV was classified as a priority pathogen by the World Health Organization (WHO) and classified as a category C bioterrorism agent by the United States, Centers for Disease Control and Prevention (CDC), as it poses an epidemic threat and has no licensed vaccines or specific antiviral therapeutics [12,16,17]. All work with authentic NiV must be carried out in Biosafety Level 4 (BSL-4) laboratories since it is a Risk Group 4 pathogen. Indeed, the need for high-containment research facilities remains a significant barrier to developing effective NiV countermeasures. These challenges pose a significant obstacle, decrease preclinical advancements, and hamper overall drug development approaches [18].

Traditional drug discovery processes begin with screening large chemical libraries using cell-based assays, which is often slow and costly. Moreover, this approach is frequently stalled due to the high rate of compound failure as leads move through the pipeline [19]. In recent years, computer-aided drug design offers a fast and cost-effective way to navigate the early stages of discovery. Molecular docking and dynamics simulation facilitate the exploration of expansive chemical spaces and aids in the identification of candidates with high predicted binding affinities. This strategy has been shown to improve early-stage success, as many compounds identified through in silico screening later demonstrate strong biological activity in cell-based assays [20,21]. Furthermore, in antiviral research, correlation between in vitro inhibitory efficacy and successful in vivo outcomes supports the reliability of this approach [22,23]. Despite the promise, integrated pipelines that combine systematic in silico screening with targeted in vitro validation remain scarce for NiV antiviral development.

Quercetin and related flavonoids have previously been proposed as NiV attachment (G) glycoprotein binders based on in silico predictions. A docking, MM-GBSA, ADME/T and MD study of 418 neem-associated phytochemicals ranked quercetin (PubChem CID 5280343) as one of the leading candidates against the NiV-G glycoprotein [24]. Of 53 natural compounds, a multi-dimensional in silico screen selected quercetin 3-galactoside among four NiV-G binders [25]. However, none of these reports included experimental testing against a henipavirus. The contribution of the present work is therefore not the computational identification of quercetin but its experimental validation in a henipavirus entry assay, together with the previously unreported synergy activity by ascorbic acid.

This study aimed to integrate an in silico screening strategy with in vitro approaches, using a recombinant Cedar virus (rCedV-NiV-B-GFP) that encodes the NiV Bangladesh strain fusion (F) and attachment (G) envelope glycoproteins together with green fluorescent protein (GFP) as a reporter gene, suitable for use at BSL-2 containment [26]. The recombinant CedV platform virus was developed using a reverse genetics approach, where the non-pathogenic CedV was rescued as a BSL-2 reagent for henipavirus research and antiviral drug discovery [27,28,29]. Because the NiV-G glycoprotein is responsible for initiating infection by binding to the host cell entry receptors, ephrin-B2 and ephrin-B3, this interaction is a potential and strategic target for therapeutic intervention [30,31,32,33]. Thus, compounds that block this specific interaction between NiV and host cells could offer promising therapeutic options. The present study aimed to develop a potential drug discovery strategy that couples high-throughput virtual screening with focused in vitro validation using the rCedV-NiV-B-GFP model system.

## 2. Results

### 2.1. Quantitative Evaluation of Ligand Bindings

In the molecular docking screening of approximately 215,000 compounds, ten candidates achieved docking scores ranging from −8.381 to −7.185 (Supplementary Table S5). The ten candidates were further evaluated using Prime MM-GBSA binding-energy estimation and protein–ligand interaction analysis. Based on the combined assessment of docking score, estimated MM-GBSA binding energy, and interactions with residues at the target binding site, four compounds were shortlisted against the NiV-G attachment glycoprotein, summarized in Table 1. Quercetin (CID 5280343) exhibited the best docking score (−8.381), followed closely by CID 2815223 (−8.241). CID 104762 and CID 25163987 showed moderately less favorable docking scores (−7.872 and −7.674, respectively). However, the MM-GBSA values are computational estimates of binding free energy rather than measurements. CID 25163987 displayed the most favorable binding free energy (−52.15 kcal/mol), followed by CID 2815223 (−43.13 kcal/mol) and quercetin (−34.22 kcal/mol). CID 104762 exhibited the weakest binding free energy (−3.48 kcal/mol), indicating poor energetic stabilization of the complex.

### 2.2. Interactions and Residue Contacts Between Protein and Ligands

**Figure 1:**
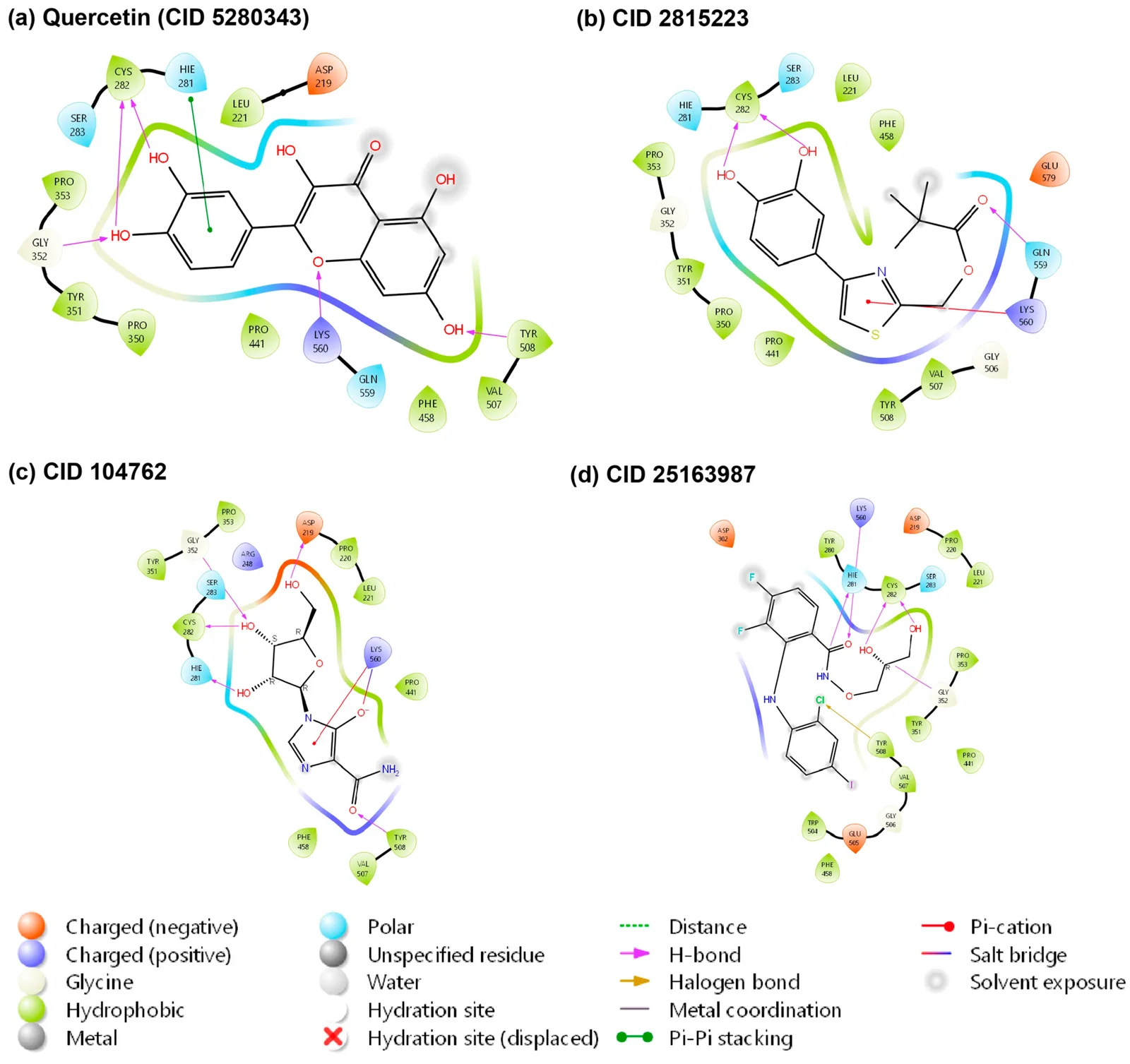
Protein–ligand interaction profiles of shortlisted compounds with NiV-G glycoprotein. Two-dimensional interaction diagrams illustrate the binding modes of NiV-G in complex with (**a**) quercetin, (**b**) CID 2815223, (**c**) CID 104762, and (**d**) CID 25163987.

Protein–ligand interactions were analyzed using two-dimensional interaction diagrams (Figure 1). All four compounds bound to the ephrin-binding region of NiV-G. Quercetin (Figure 1a) formed hydrogen bonds with Gly352, Lys560, Tyr508, and Cys282 (two bonds), and a π-interaction with His281. CID 2815223 (Figure 1b) established hydrogen bonds with Gln559 and Cys282 (two bonds), and a π–cation interaction with Lys560. CID 104762 (Figure 1c) formed hydrogen bonds with Cys282 and Gly352, and π-stacking interactions with Lys560, along with contacts at His281, Asp219, and Tyr508. CID 25163987 (Figure 1d) showed the most extensive network, with hydrogen bonds at Cys282 (two bonds), His281, Gly352, and Lys560, and a halogen bond with Tyr508.

### 2.3. Pharmacokinetics and Drug-likeness Evaluation

The in silico predicted ADME/T properties of the four compounds, generated using Schrödinger’s QikProp module, are summarized in Table 2. CID 2815223 showed high predicted oral absorption (HOA ≈ 89%), moderate lipophilicity (QPlogPo/w = 2.759), and acceptable solubility (QPlogS = −4.677), though it exhibited a mild cardiotoxicity risk (QPlogHERG = −5.286). CID 25163987 also demonstrated high oral absorption (HOA ≈ 88%) and greater lipophilicity (QPlogPo/w = 2.962), but with lower solubility (QPlogS = −4.922). Despite its strong membrane permeability, it had a higher cardiotoxicity risk (QPlogHERG = −5.667) and contained a reactive functional group. Quercetin showed moderate predicted oral absorption (~51.6%) and favorable predicted solubility (QPlogS = −2.901). Its QPlogHERG value of −5.097 falls slightly below the −5 threshold, indicating a potential hERG-related concern. CID 104762 exhibited the lowest absorption (HOA ≈ 20.9%) but the highest solubility (QPlogS = −1.483) and the lowest cardiotoxicity risk (QPlogHERG = −3.451). To assess the reliability of the four shortlisted candidates beyond docking and MM-GBSA scoring, we screened each structure for known assay-interference and undesirable substructure liabilities. Notably, quercetin and CID 2815223, the two candidates with the most favorable MM-GBSA binding free energies, were both flagged under the PAINS catechol_A alert, which was independently corroborated by the Brenk catechol alert in each case, reflecting the catechol-type substructure present in both compounds. CID 104762 returned no PAINS or Brenk alerts. CID 25163987 returned no PAINS alerts but was flagged for several Brenk substructures (two imine groups, an aryl iodide, and an oxygen–nitrogen single bond), substructures generally considered undesirable for drug development due to potential reactivity or metabolic liability, though not classified as PAINS-type assay-interference patterns. All four compounds satisfied REOS property-based filter criteria. Given that two of our top candidates carry a recognized PAINS liability, we address the implications of this finding for interpretation of our antiviral data directly in Section 3 (Discussion).

### 2.4. Molecular Dynamics (MD) Simulation

#### 2.4.1. Root Mean Square Deviation (RMSD)

**Figure 2:**
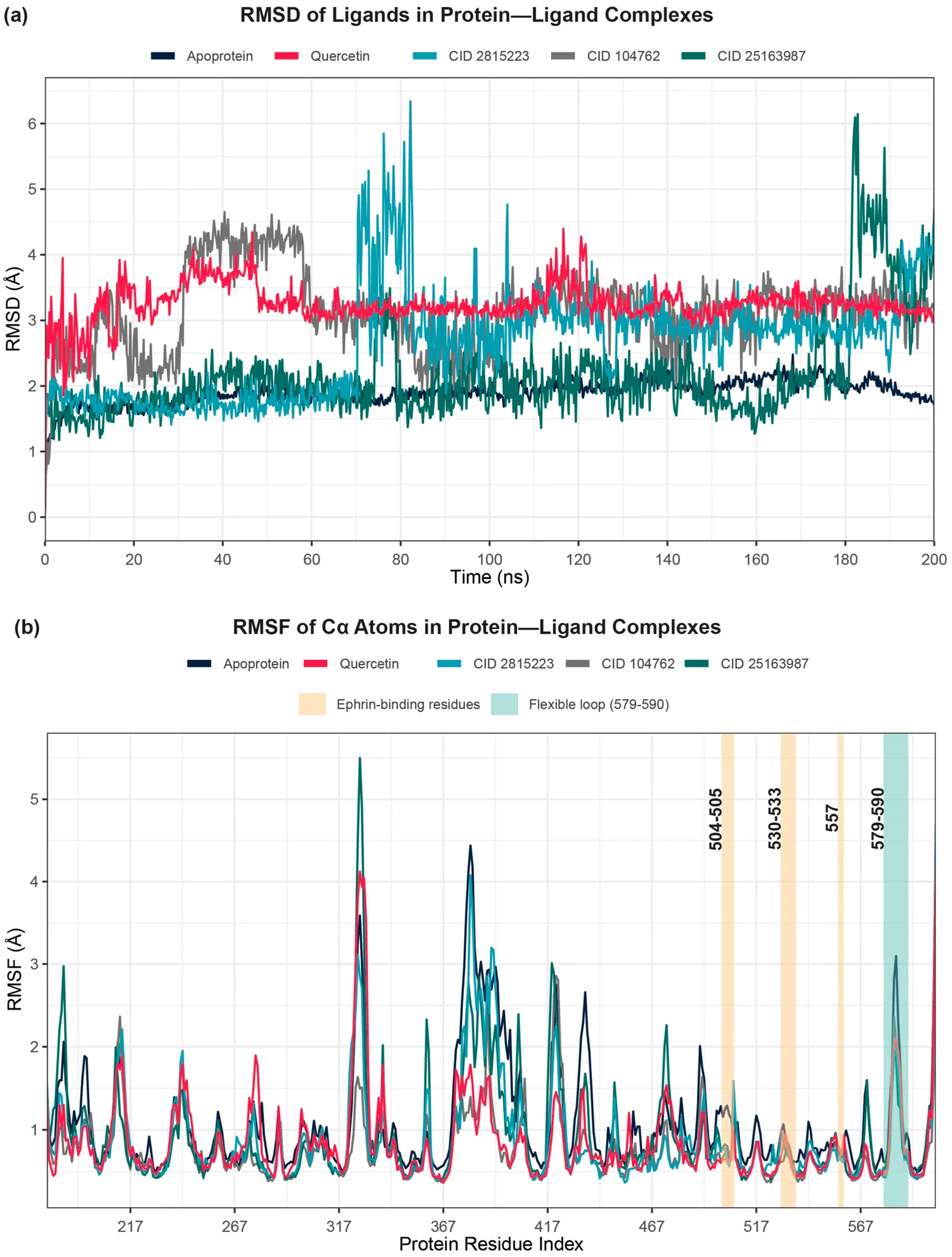
Molecular dynamics-based stability analysis of NiV-G–ligand complexes. (**a**) Root mean square deviation (RMSD) trajectories of the NiV-G apoprotein and ligands in protein–ligand complexes over 200 ns of molecular dynamics simulations. (**b**) Root mean square fluctuation (RMSF) profiles of Cα atoms of NiV-G residues in complex with the four shortlisted ligands and NiV-G apoprotein, indicating residue-level flexibility throughout the simulation period.

**Figure 3:**
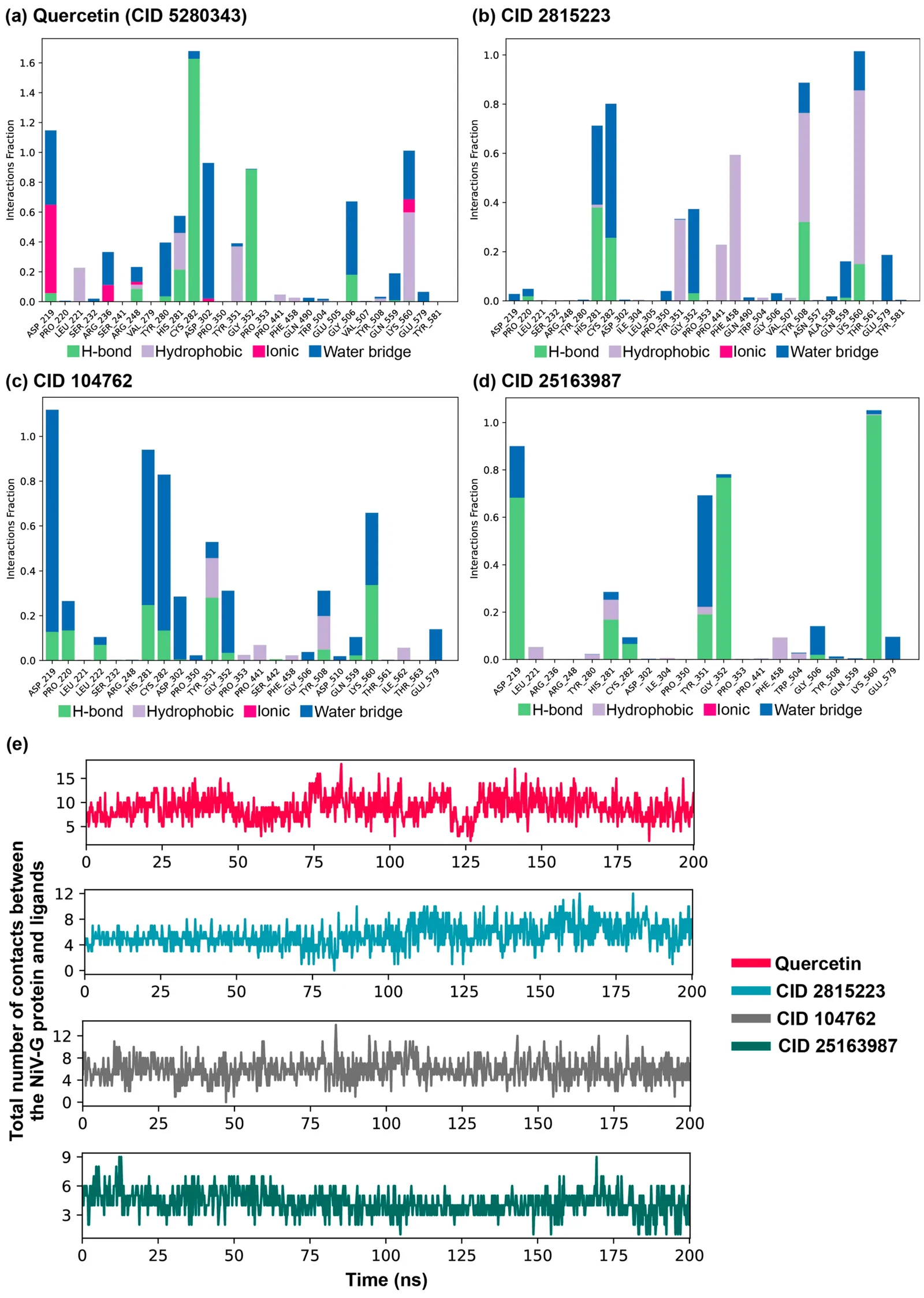
Protein–ligand contact analysis during molecular dynamics simulations. Protein–ligand contact histograms showing the frequency and type of interactions between NiV-G and (**a**) quercetin, (**b**) CID 2815223, (**c**) CID 104762, and (**d**) CID 25163987 over 200 ns simulations. Interaction fractions > 1.0 indicate residues involved in multiple simultaneous contacts. (**e**) Timeline plot depicting the total number of contacts maintained between NiV-G and each ligand throughout the simulation.

The structural stability of the NiV-G protein in complex with the four ligand candidates was evaluated using RMSD analysis over a 200 ns MD simulation (Figure 2a). The unbound NiV-G apoprotein maintained a stable reference conformation, with a baseline RMSD of approximately 2.0 Å throughout the simulation. Among the candidates, the quercetin complex exhibited the lowest variance, with RMSD fluctuations modestly ranging from 3.25 ± 0.3 Å, indicating that the ligand remained well-accommodated in the binding site with minimal deviation from the initial structure. In contrast, the CID 2815223 complex showed more variability, with RMSD values rising above 5 Å at times, suggesting greater flexibility in the binding site. The complexes with CID 104762 and CID 25163987 exhibited the largest RMSD deviations, indicating substantial structural instability and greater conformational changes during the simulation. Because the quercetin-NiV-G RMSD, although the most stable among the four candidates, remained above 3 Å for much of the simulation, we further examined the protein–ligand interaction pattern across the stable, production-phase portion of the trajectory (100–200 ns) by clustering per-frame contact fingerprints (hydrogen bonds, hydrophobic, ionic, metal/ion-bridged, π-cation, π-π, and water-bridged contacts). This identified three recurring interaction states, each populated throughout the 100–200 ns window rather than confined to a single time segment. The dominant state, accounting for 47.8% of production-phase frames, was defined by persistent hydrogen bonds with Cys282 and Gly352 (present in 98% of its frames), together with a water-bridged interaction with Asp302 (82%) and an ion-bridged contact with Asp219 (50%). Two additional recurring states retained the Cys282 hydrogen bond as an essentially invariant anchor (present in >90% of frames in every state) while alternating additional contacts: a π-cation interaction with Lys560 and hydrogen bond with His281 in one state (32.1% of frames), and an additional hydrogen bond with Gly506 in the other (20.1% of frames). This indicates that, despite the RMSD fluctuation noted above, quercetin does not adopt a single static pose but toggles among a small number of recurring, structurally related interaction modes, all anchored by the Cys282 hydrogen bond identified as the strongest persistent contact in Figure 3e.

#### 2.4.2. Root Mean Square Fluctuation (RMSF)

RMSF analysis was conducted to evaluate the dynamic impact of ligand binding on the NiV-G glycoprotein. At the same time, all four complexes exhibited a high degree of RMSF profile similarity, with fluctuations of Cα atoms consistently remaining below 3 Å (with the exception of a few minor peaks) (Figure 2b). Quercetin emerged as the superior stabilizer of the NiV-G glycoprotein. It specifically restricts the movement of critical residues involved in ephrin B2/B3 recognition (Trp504, Glu505, Gln530, Thr531, Ala532, Glu533, and Asn557) and the flexible loop (residues 579–590) to a narrow 1–2 Å range. CID 104762 maintained a remarkably stable profile below 3 Å, and even showed lower baseline mobility than quercetin in certain regions. In contrast, CID 2815223 showed localized flexibility in the 377–387 residue stretch, and the CID 25163987 complex mirrored its RMSD profile by exhibiting higher fluctuations (RMSF values) than other leads, though within a favorable range.

#### 2.4.3. Protein–Ligand Contact Mapping

Protein–ligand contact histograms were used to analyze the interactions between NiV-G and the four candidate ligands during 200 ns MD simulations (Figure 3). Interaction fractions greater than 1.0 indicate residues involved in multiple interactions. Quercetin (Figure 3a) formed strong, persistent hydrogen bonds with CYS282 (fraction > 1.6) and GLY352 (~0.85), as well as ionic and water-bridged interactions at ASP219 (~1.1) and ASP302 (~0.9), maintaining high occupancy throughout the simulation. Moreover, quercetin also maintained contact with GLY506 (~0.7) and LYS560 (~1.1) through water-bridge and hydrophobic interactions, respectively, suggesting its potential to block nearby key residues involved in ephrin B2/B3 recognition, such as Trp504, Glu505 and Asn557. CID 25163987 (Figure 3d) also showed stable interactions, with hydrogen bonds at LYS560 (fraction > 1.0), ASP219, and GLY352. CID 2815223 (Figure 3b) maintained stability through hydrophobic and water-bridged interactions at TYR508 (~0.9) and LYS560 (~1.0), though it lacked the extensive hydrogen bonding seen in quercetin and CID 25163987. CID 104762 (Figure 3c) displayed weak interactions, reflecting structural instability.

#### 2.4.4. Protein–Ligand Contact Timeline

The contact timeline analysis (Figure 3e) showed that quercetin maintained a highly stable interaction profile of 9–10 contacts throughout the 200 ns simulation. While CID 2815223 achieved peak contacts of up to 12, it was less consistent, fluctuating down to four. CID 25163987 and CID 104762 displayed lower interaction profiles, fluctuating between 3–6 and 4–8 contacts, respectively, indicating reduced stability compared to the lead compound. Consequently, quercetin represents the most promising binder in terms of contact persistence.

### 2.5. Selection of Lead Candidate for In Vitro Validation

Among the 215,000 compounds screened, quercetin was identified as the most promising candidate. While CID 25163987 showed a slightly more favorable MM-GBSA binding free energy, quercetin demonstrated a superior docking score (−8.381) and, more importantly, a highly stable interaction profile throughout the 200 ns molecular dynamics simulation, maintaining 9–10 consistent contacts with the NiV-G glycoprotein. Therefore, quercetin was selected for subsequent cell-based experimental validation.

### 2.6. Virus Titer of rCedV-NiV-B-GFP

The infectious titer of the rCedV-NiV-B-GFP was determined by plaque assay on Vero cells. Distinct, well-defined plaques were observed following 4 days of incubation under carboxymethylcellulose overlay. Plaque numbers decreased proportionally with increasing serial dilutions. Based on plaque counts, the viral titer of the rCedV-NiV-B-GFP was calculated to be 3.7 × 10^8^ PFU/mL. This titer was subsequently used to standardize the MOI for downstream neutralization and antiviral assays.

### 2.7. Cytotoxic Effects of Quercetin on Vero Cells Using MTS Assay

**Figure 4:**
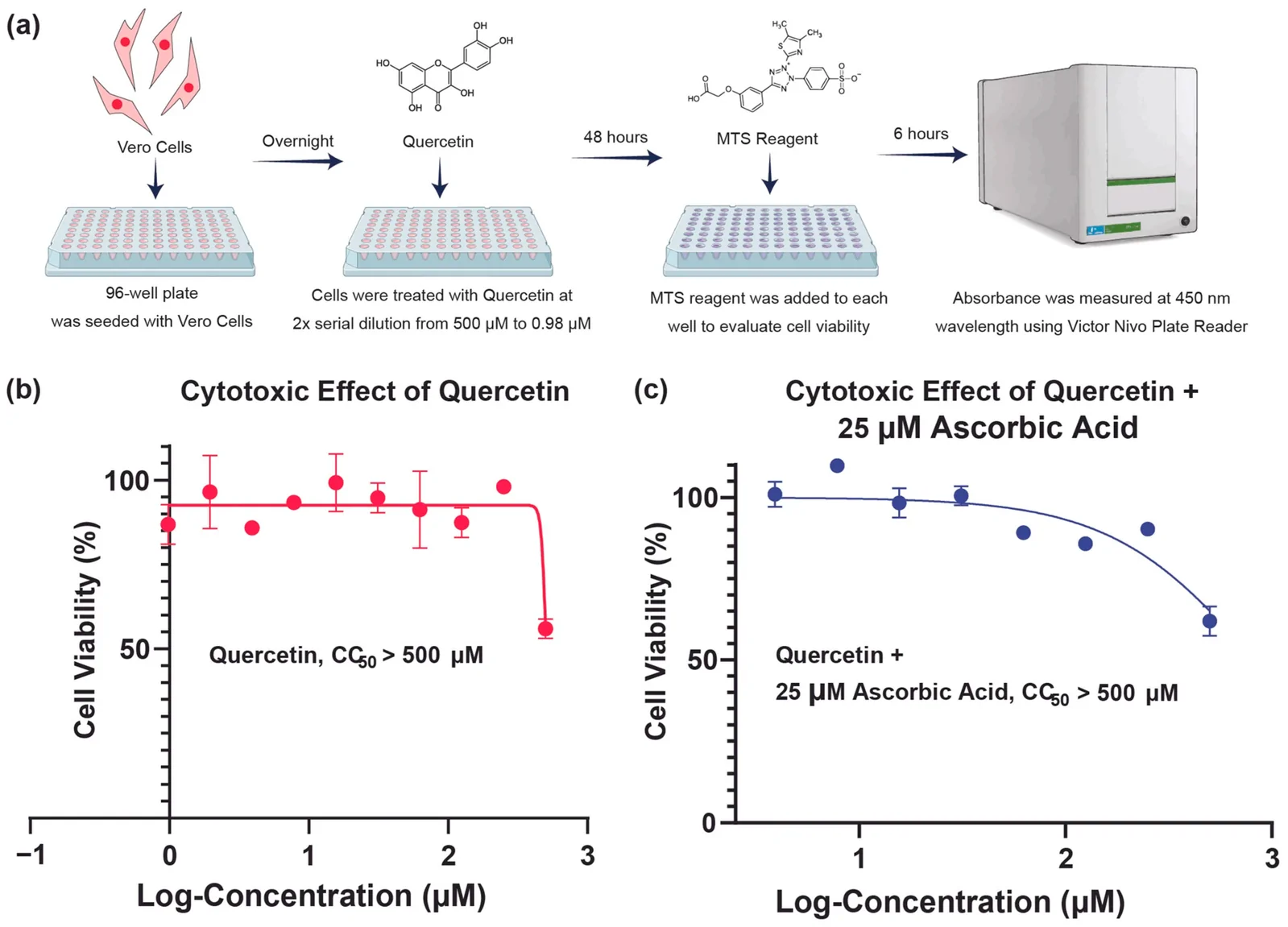
Evaluation of quercetin cytotoxicity on Vero cells. (**a**) Schematic workflow of the MTS cytotoxicity assay. Vero cells were seeded in a 96-well plate and incubated overnight to achieve 80–90% confluency. Cells were then treated with quercetin at 2-fold serial dilutions (500 µM to 0.98 µM) and incubated for 48 h. Following incubation, 20 µL of MTS reagent was added to each well, and cell viability was assessed by measuring absorbance at 450 nm after 6 h. (**b**) Dose–response curve illustrating the percentage of cell viability relative to the Log-concentration of quercetin. Viability was calculated by normalizing absorbance data against cell and media controls. The calculated CC_50_ for quercetin on Vero cells was more than 500 µM. Error bars represent the standard deviation of three technical replicate wells. (**c**) Dose–response curve illustrating the percentage of cell viability relative to the Log-concentration of quercetin + 25 μM ascorbic acid. Viability was calculated by normalizing absorbance data against cell and media controls. The calculated CC_50_ for quercetin + 25 μM ascorbic acid on Vero cells was more than 500 µM. Error bars represent the standard deviation of three technical replicate wells.

To determine the safety profile of quercetin, its cytotoxic effect on Vero cells was evaluated using an MTS colorimetric assay (Figure 4a). Quercetin was applied to Vero cells in two-fold serial dilutions ranging from 500 µM to 0.98 µM and was incubated for a period of 48 h. Cell viability was quantified by measuring the absorbance at 450 nm. The percentages of cell viability were calculated by comparing treated wells to cell and media controls. The assay showed that quercetin maintained high cell viability across the majority of the tested range (Figure 4b). The 50% cytotoxic concentration (CC_50_) for quercetin was calculated to be more than 500 µM. Similarly, quercetin in combination with 25 µM ascorbic acid also did not reduce cell viability below 50% within the tested concentration range, resulting in a CC50 of >500 µM in Vero cells.

### 2.8. Quercetin Antiviral Efficacy Against rCedV-NiV-B-GFP

**Figure 5:**
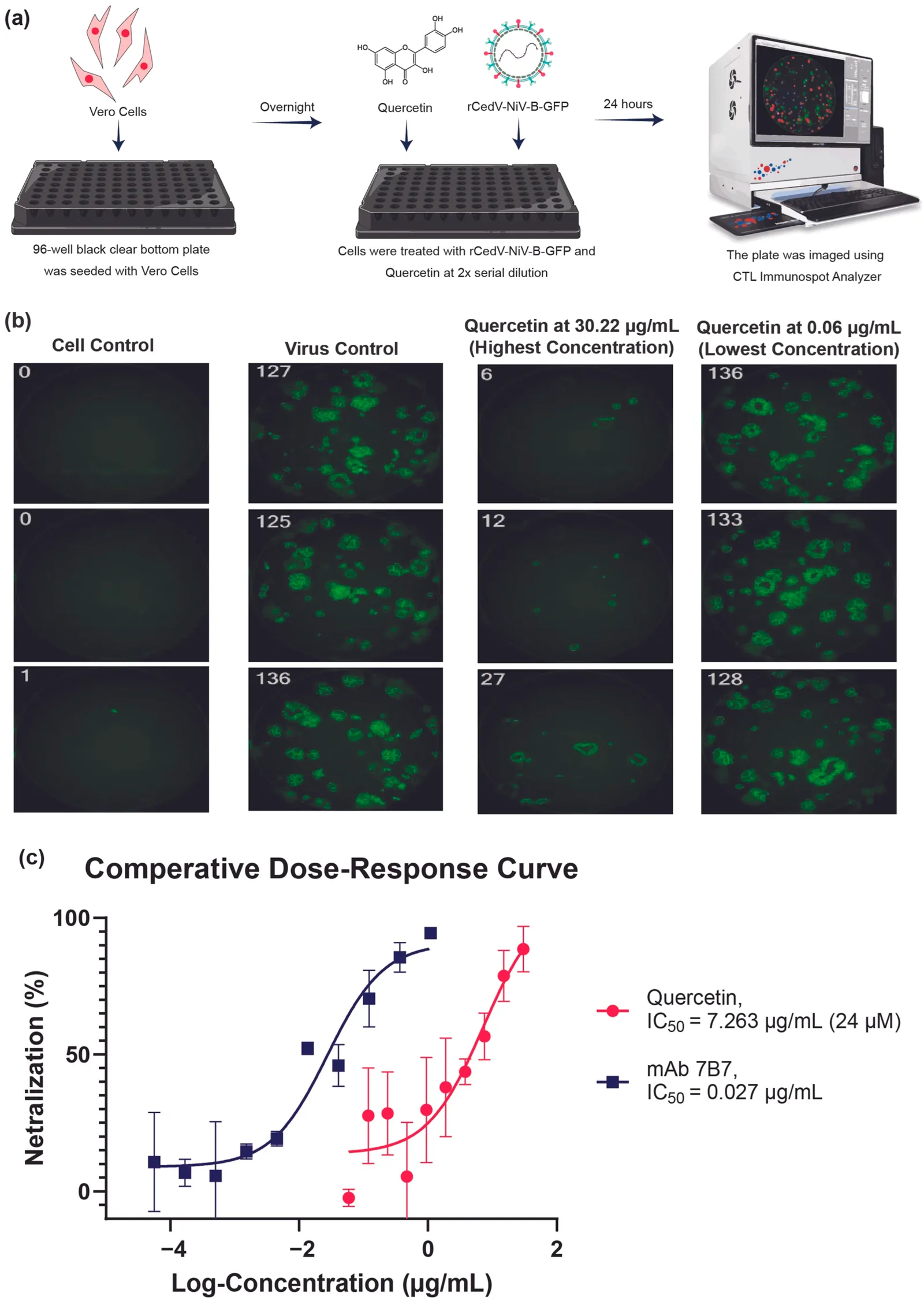
Antiviral activity of quercetin against NiV using rCedV-NiV-B-GFP dependent FRNT. (**a**) Schematic overview of the FRNT. Vero cells were seeded in a 96-well black clear-bottom plate and incubated overnight. Cells were then treated with quercetin at 2-fold dilutions and GFP-expressing rCedV encoding NiV glycoproteins. Following 24 h of incubation, the plate was fixed, washed, imaged using the CTL Immunospot Analyzer, and GFP-positive foci were quantified. (**b**) Representative fluorescence microscopy images of FRNT wells. Images show GFP-positive foci (green) for the cell control (uninfected), the virus control (rCedV-NiV-B-GFP only), and wells treated with the highest (30.22 μg/mL) and lowest (0.06 μg/mL) concentrations of quercetin. The number in the upper left corner of each image represents the automatically counted number of foci. A marked reduction in foci count in the presence of 30.22 μg/mL quercetin compared to the virus control was observed. (**c**) Dose-dependent neutralization curve for quercetin and positive control (mAb 7B7). The neutralization percentage is plotted against the Log concentration (μg/mL) for quercetin (red circles) and the positive control mAb 7B7 (blue squares). Data were fitted to a non-linear regression curve to determine the IC50. The calculated IC_50_ for quercetin was 7.263 μg/mL (24 μM; 95% CI: 2.30–28.30 µg/mL) and 0.027 μg/mL (95% CI: 0.0108–0.0735 µg/mL) for the mAb 7b7. Error bars represent the standard deviation of three technical replicate wells.

To assess the antiviral potential of quercetin against NiV, a FRNT utilizing rCedV-NiV-B-GFP was conducted. Quercetin was tested across concentrations ranging from 30.22 μg/mL to 0.06 μg/mL with a 2-fold serial dilution (Figure 5a). The treatment demonstrated a sigmoidal pattern of dose-dependent reduction in GFP-positive foci. At the highest concentration tested (30.22 μg/mL), the average foci count was 15, compared with an average of 129.33 foci in the virus-only control (Figure 5b). The calculated IC_50_ value for quercetin was 7.263 μg/mL (24 μM; 95% CI: 2.30–28.30 µg/mL) (Figure 5c). This activity was compared to the positive control, mAb 7B7, which exhibited an IC_50_ value of 0.027 μg/mL (95% CI: 0.0108–0.0735 µg/mL). By correlating these results with the cytotoxicity data, the Selectivity Index (SI) for quercetin was determined to be >20.8.

### 2.9. Synergistic Effect of Quercetin and Ascorbic Acid on Neutralizing rCedV-NiV-B-GFP

**Figure 6:**
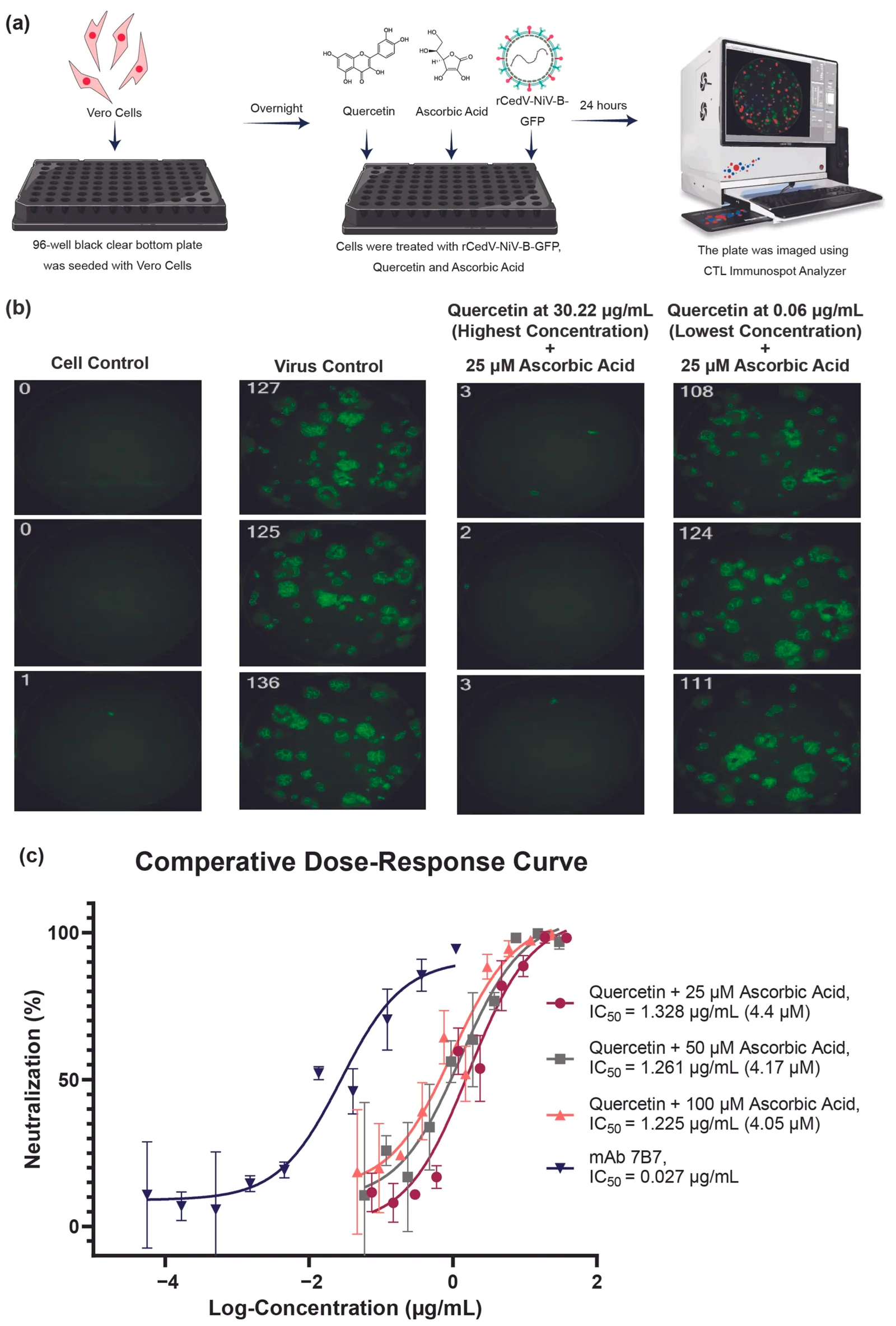
Enhanced antiviral activity of quercetin in combination with ascorbic acid against rCedV-NiV-B-GFP. (**a**) Schematic representation of the FRNT workflow. Vero cells were seeded in 96-well black clear-bottom plates and incubated overnight to achieve 80–90% confluency. Cells were subsequently treated with a mixture of quercetin (at 2-fold serial dilutions), a fixed concentration of 25 μM ascorbic acid, and rCedV-NiV-B-GFP at a MOI of 0.05. After a 24 h incubation period, plates were fixed with 4% formaldehyde and imaged using the CTL ImmunoSpot Analyzer to quantify GFP-positive foci. (**b**) Representative fluorescence microscopy images of FRNT wells showing GFP-positive foci (green) for cell control (uninfected), virus control (rCedV-NiV-B-GFP only), and combination treatments at the highest (30.22 μg/mL quercetin + 25 μM ascorbic acid) and lowest (0.06 μg/mL quercetin + 25 μM ascorbic acid) tested concentrations. The numerical value in the upper-left corner of each well indicates the automatically counted number of discrete foci. (**c**) Comparative dose–response neutralization curves for the quercetin–ascorbic acid combinations and the positive control monoclonal antibody mAb 7B7 (blue squares). Data were fitted using a four-parameter log-logistic regression model to determine IC_50_ values. The combination treatment exhibited IC_50_ values of 1.328 μg/mL (4.4 μM), 1.261 μg/mL (4.17 μM), and 1.225 μg/mL (4.05 μM) for 25 µM, 50 µM, and 100 µM ascorbic acid with quercetin, respectively. The mAb 7B7 control showed an IC_50_ of 0.027 μg/mL. Error bars represent the standard deviation of three technical replicate wells.

Quercetin is readily oxidized under physiological and cell culture conditions [34]. Based on the antioxidant properties of ascorbic acid, we hypothesized that its inclusion might help preserve quercetin activity. To assess the combined effect of quercetin and ascorbic acid in terms of neutralizing rCedV-NiV-B-GFP, an FRNT was performed using quercetin at concentrations ranging from 30.22 μg/mL to 0.06 μg/mL (2-fold serial dilution) and a fixed concentration of 25 μM ascorbic acid (Figure 6a). Representative fluorescence microscopy images demonstrated a substantial decrease in GFP-positive foci in wells treated with the combination compared to the virus control (Figure 6b). At the highest tested concentration (30.22 μg/mL quercetin + 25 μM ascorbic acid), the automatically counted foci dropped to an average of 2.67, compared to counts exceeding 125 in the virus-only control. The IC_50_ for quercetin alone was calculated at 7.26 µg/mL (24.0 µM; 95% CI: 2.30–28.30 µg/mL, or 7.6–93.6 µM). However, the quercetin + 25 µM ascorbic acid combination achieved an IC_50_ of 1.328 µg/mL (4.39 µM; 95% CI: 0.8652–2.050 µg/mL, or 2.9–6.8 µM), representing a more than 5-fold increase in potency (Figure 6c). Higher concentrations of ascorbic acid further enhanced this effect, with IC_50_ values reaching 1.261 µg/mL (4.17 µM; 95% CI: 0.6346–2.499 µg/mL, or 2.1–8.3 µM) at 50 µM and 1.225 µg/mL (4.05 µM; 95% CI: 0.6492–2.299 µg/mL, or 2.2–7.6 µM) at 100 µM.

**Figure 7:**
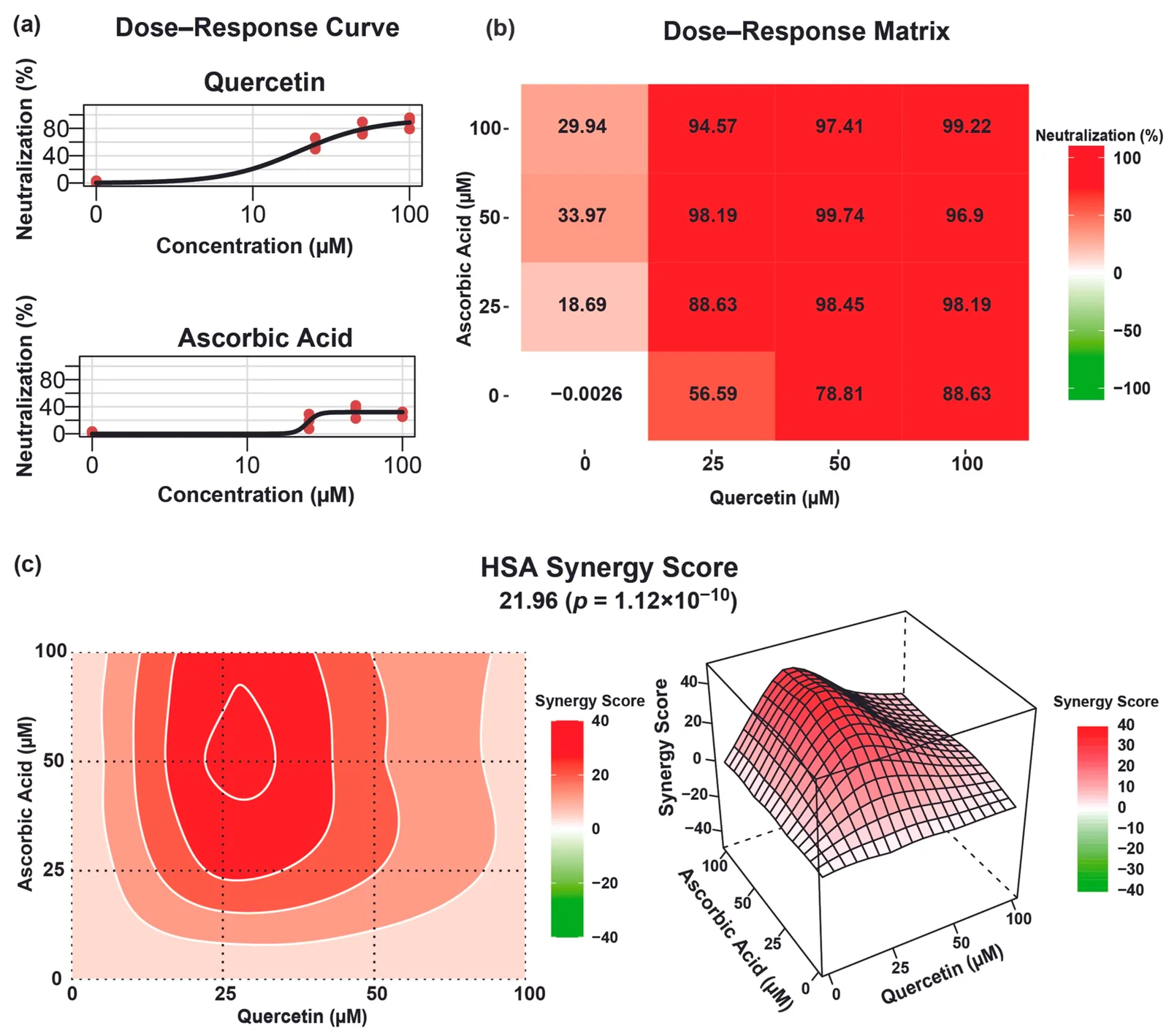
Synergy analysis of quercetin and ascorbic acid combinations using the HSA model. (**a**) Individual dose–response curves for quercetin and ascorbic acid against rCedV-NiV-B-GFP. (**b**) Dose–response matrix representing the mean neutralization percentage for various concentration combinations of quercetin and ascorbic acid. (**c**) Synergy contour and three-dimensional surface plots representing major synergy hotspots.

Single-agent dose–response analysis showed that ascorbic acid alone exhibited limited neutralization activity, reaching 29.94% at 100 μM concentration. Quercetin alone reached 88.63% neutralization at 100 μM (Figure 7a). However, the quercetin–ascorbic acid combination showed enhanced neutralization compared with the individual compounds under the tested conditions. The concentration pair (50 μM quercetin + 50 μM ascorbic acid) resulted in near-complete neutralization of 99.74% (Figure 7b). Quantitative synergy analysis using the Highest Single Agent (HSA) model revealed a synergistic interaction (Figure 7c). The HSA synergy score was 21.96 (*p* = 1.12 × 10^−10^). At quercetin concentrations of 25–50 µM, a pronounced synergy hotspot was found by analyzing the synergy contour and three-dimensional surface plots.

## 3. Discussion

NiV is a highly pathogenic zoonotic henipavirus responsible for recurrent outbreaks across South Asia, particularly in Bangladesh and India. Recent data from outbreaks in Bangladesh continue to demonstrate high clinical severity, with reported case fatality rates ranging from 70% to 100% [11,35]. Despite high CFR in each outbreak, medical countermeasures are limited, and there are no approved vaccines or antiviral therapeutics are available for NiV [36]. In this study, we utilized an integrated drug discovery pipeline that combined high-throughput virtual screening, molecular dynamics (MD) simulations, and cell-based neutralization assays. This integrated approach identified quercetin as a potential inhibitor of the NiV attachment glycoprotein (NiV-G).

The in silico analysis targeted the receptor-binding site of NiV-G (PDB: 3D11), which interacts with host ephrin-B2/B3 receptors. Disruption of the interaction between NiV-G and host ephrin-B2/B3 receptor has been shown to effectively block membrane fusion and viral entry, thereby preventing productive infection [31]. More than 215,000 drug-like compounds were virtually screened against the ephrin-B2/B3 binding site of NiV-G, and the binding free energy of the compounds was estimated using the Molecular Mechanics/Generalized Born Surface Area (MM-GBSA) algorithm. Quercetin was identified with the most favorable docking score (−8.381). Although some compounds showed better MM-GBSA scores than quercetin (MM-GBSA: −34.22 kcal/mol), such as CID 2815223 (MM-GBSA: −43.13 kcal/mol) and CID 25163987 (MM-GBSA: −52.15 kcal/mol), In a 200 ns MD simulation, quercetin showed the most promising interaction at the target site of the NiV-G glycoprotein. MD trajectory analysis showed that the average root mean square deviation (RMSD) for quercetin in the quercetin–NiV-G complex is 3.25 ± 0.3 Å, suggesting a stable interaction throughout the simulation [37]. Root mean square fluctuation (RMSF) analysis showed that quercetin restricts the movement of critical residues involved in ephrin B2/B3 recognition (Trp504, Glu505, Gln530, Thr531, Ala532, Glu533, and Asn557) and the flexible loop (residues 579–590) to a narrow range (1.10 ± 0.49 Å) compared to the NiV-G glycoprotein (1.51 ± 0.80 Å) (Figure 2b). Notably, restricting the conformational flexibility of the 579–590 loop is important because this region has previously been reported to undergo structural rearrangement upon ephrin engagement [38]. Such stabilization likely impedes the conformational adaptability required for high-affinity receptor binding, providing a plausible structural basis for a potential entry-inhibition mechanism; however, this was not directly tested in this study. Protein–ligand contact analysis showed that quercetin maintained multiple interactions with NiV-G throughout the 200 ns simulation (Figure 3e), with a generally higher contact frequency than the other shortlisted compounds. Although the number and identity of individual contacts fluctuated over time, several interactions recurred during the trajectory. Overall, quercetin exhibited a persistent interaction profile, indicating that it remained associated with the binding region over the simulated timescale. In silico pharmacokinetic and drug-likeness evaluation further supported quercetin as a viable lead compound (Table 2). Quercetin satisfied Lipinski’s Rule of Five and exhibited favorable solubility and safety parameters. Its predicted QPlogHERG value of −5.097 falls slightly below the −5 threshold commonly associated with potential hERG liability, similar to two of the other three compounds. These predictions are approximate, and experimental hERG testing is required before any conclusions regarding cardiac safety can be drawn.

Collectively, the in silico analyses presented here identified quercetin as a promising NiV entry inhibitor. Quercetin is a well-characterized flavonoid with broad-spectrum antiviral activity reported against multiple enveloped RNA viruses, including influenza virus, dengue virus, Zika virus, and coronaviruses, where it has been shown to interfere with viral entry, replication, and protease activity [39,40]. Cell-based experiments using rCedV-NiV-B-GFP confirmed the in silico-predicted antiviral activity of quercetin; however, the activity of quercetin against authentic NiV requires confirmation under BSL-4 containment [26].

Quercetin demonstrated a dose-dependent neutralization of the virus with an IC_50_ of 24 µM (7.263 μg/mL). While this potency is lower than the positive control monoclonal antibody mAb 7B7, its activity is notable for a naturally occurring small molecule. This finding aligns with previous reports of flavonoid-mediated entry inhibition in paramyxoviruses and other enveloped viruses [41,42,43,44], although the specific stage of the viral life cycle affected by quercetin in the present study was not directly determined. Moreover, quercetin exhibited minimal cytotoxicity in Vero cells, with a CC_50_ > 500 μM. The Selectivity Index (SI) for quercetin was >20.8. An SI greater than 10 is generally considered the benchmark for a promising antiviral lead [45]. A SI value >20.8 indicating that quercetin can achieve therapeutic efficacy at concentrations well below its toxic threshold.

A primary discovery of this study is the enhancement of quercetin’s antiviral efficacy in the presence of ascorbic acid. Quercetin is readily oxidized under physiological and cell culture conditions, which leads to loss of bioactivity. One possible explanation is that ascorbic acid, a potent antioxidant, may preserve quercetin in its reduced, bioactive form [46]. While ascorbic acid alone showed no antiviral activity (Figure S1), just 25 μM ascorbic acid with quercetin markedly reduced the IC_50_ to 4.4 μM (1.328 μg/mL), a more than fivefold increase in potency. In the presence of 25 μM ascorbic acid, the SI value of quercetin was >113.64. This synergy resulted in near-complete viral neutralization (99.74%) at 50 µM concentrations of quercetin (Figure 7b). Statistical validation via the Highest Single Agent (HSA) model verified a potent synergistic relationship, yielding an average synergy score of 21.96 (Figure 7c). An HSA mean synergy score greater than 10 indicates that the observed effect exceeds simple additive activity [47]. Beyond its in vitro antiviral activity, quercetin is a well-characterized and widely available compound. However, its commercial availability does not establish its in vivo antiviral feasibility. Human pharmacokinetic studies indicate that systemic quercetin concentrations after oral administration are generally below the 4.4–24 µM antiviral range observed in the present study, although enhanced formulations and repeated dosing can substantially increase systemic exposure. Therefore, further pharmacokinetic studies, improved formulation strategies, and in vivo antiviral evaluation are required to determine whether therapeutically relevant concentrations can be achieved at target tissues [48].

While findings of this study provide strong evidence that quercetin inhibits NiV infection, several limitations should be acknowledged regarding the mechanism responsible for this activity. Our neutralization assay design, in which quercetin remains in contact with both virus and cells throughout the full 24 h infection period, without a wash step separating attachment from later stages of infection, demonstrates that quercetin reduces productive NiV infection but cannot, on its own, establish that this occurs specifically at the viral entry step. Quercetin’s known broad-spectrum antiviral activity against structurally unrelated viruses raises the alternative possibility that some of its effect could reflect a non-specific or cell-directed antiviral state rather than direct interference with NiV-G-mediated entry. Our computational data (docking at the ephrin-B2/B3 binding interface and MD-based restriction of the ephrin-recognition residues, Figure 2b) provide a plausible structural basis for a virus-specific, entry-stage mechanism, but direct biophysical confirmation of binding (e.g., microscale thermophoresis or nanoDSF) and stage-resolved time-of-addition experiments will be required to confirm this mechanism and rule out a cell-directed component. We consider this the most important next step for validating quercetin as a NiV entry inhibitor. The antiviral assays were performed using a surrogate virus system rather than authentic NiV. Therefore, confirmation of these findings in BSL-4 settings will be essential. In addition, a formal validation of the docking protocol, such as redocking of a co-crystallized ligand or comparison with a known NiV-G small-molecule complex, was not performed. Furthermore, only a single 200 ns MD trajectory was generated for each protein–ligand system, without independent replicate simulations. Therefore, the docking and MD results should be interpreted as computational predictions that require further validation. Additionally, although quercetin exhibits promising in vitro efficacy, its pharmacokinetics, metabolic stability, and antiviral activity require further investigation in in vivo settings. Quercetin’s chemical instability is a relevant limitation for its therapeutic development. Its catechol B-ring undergoes auto-oxidation under physiological conditions (neutral-to-mildly alkaline pH, presence of trace transition metals, and oxidative extracellular/intracellular environments), generating quinone and semiquinone species with diminished antioxidant and, based on our findings, likely diminished antiviral activity. This susceptibility to oxidation, rather than intrinsic potency alone, may partly explain quercetin’s comparatively modest IC_50_ relative to mAb 7B7, and is consistent with the marked potency increase we observed upon co-administration with the antioxidant ascorbic acid. For therapeutic development, strategies such as chemical modification (e.g., methylation or glycosylation to yield more stable derivatives such as rutin or isorhamnetin), nanoparticle or liposomal encapsulation, or co-formulation with stabilizing antioxidants should be evaluated. Future studies should include formulation optimization and evaluation in relevant animal models. A related consideration is that two of the four shortlisted candidates, quercetin and CID 2815223, notably the two compounds with the most favorable predicted MM-GBSA binding free energies, carry a catechol-type substructure flagged by both the PAINS and Brenk structural alert filters (Section 2.3). Catechol-type PAINS liabilities are most consequential in assay formats vulnerable to redox cycling (where auto-oxidation of the catechol to a reactive quinone generates hydrogen peroxide that directly corrupts the reporter chemistry) or metal chelation (interfering with metalloenzyme active sites); this is why PAINS filters were originally curated primarily from AlphaScreen and luciferase-coupled high-throughput screens. Our primary antiviral readout is a fixed 24 h endpoint, fluorescence-microscopy-based count of GFP-positive foci, which does not depend on an enzymatic or redox-coupled signal-generating step, making it comparatively less susceptible to this specific artifact mechanism than the assay formats from which the PAINS filters were derived. We further note that quercetin’s dose–response curve was sigmoidal with a clearly defined plateau (Figure 6) rather than the flat, erratic, or non-saturating profile typically associated with aggregation-based or redox-cycling false positives. Moreover, quercetin showed no measurable cytotoxicity up to the highest concentration tested (CC50 > 500 µM), arguing against widespread non-specific cellular redox stress as an explanation for its antiviral signal. We also acknowledge the alternative possibility that Glide MM-GBSA scoring may itself be biased toward catechol-containing ligands, since their multiple adjacent hydroxyl groups can artificially inflate predicted hydrogen-bonding and polar solvation terms; the convergence of quercetin’s favorable docking/MM-GBSA score with its experimentally confirmed antiviral activity is therefore reassuring but not conclusive proof of a specific, non-artifactual binding mode. A dedicated counter-screen, for example, a detergent-based aggregation control, or an orthogonal non-fluorescent antiviral readout, would further strengthen confidence in a specific mechanism and represents an important direction for follow-up validation of both quercetin and CID 2815223. Quercetin was selected for experimental validation from among the four computationally prioritized candidates based on its superior MD stability, most extensive and persistent protein–ligand contact profile, and favorable predicted ADMET properties, together with its accessibility as an inexpensive, well-characterized natural compound. Experimental evaluation of the remaining three candidates (CID 2815223, CID 104762, and CID 25163987) was beyond the scope of the present study and is planned as immediate follow-up work to determine whether their computationally predicted binding stability translates into antiviral activity.

## 4. Materials and Methods

### 4.1. Preparation of NiV-G Glycoprotein for Structural Analysis

The receptor unbound pre-fusion structure of the NiV attachment G glycoprotein was retrieved from the Protein Data Bank (PDB) (https://www.rcsb.org/; accessed on 13 November 2024), using the PDB ID 3D11. The structure was determined by X-ray crystallography at 2.31 Å resolution [49]. The deposited structure contains a single protein chain (chain A), which was used throughout this study. All N-linked glycan residues were removed prior to protein preparation. Crystallographic water molecules located more than 5.0 Å from the protein were removed [50]. To refine the structure, Schrodinger’s Protein Preparation Wizard was used. Bond orders were assigned based on the Chemical Component Dictionary, hydrogen atoms were added, missing side chains were completed using Prime, disulfide bonds were generated, and zero-order bonds were assigned to ions where appropriate. Protein termini were not capped, and missing loops were not modeled. Ionization states of hetero groups were assigned using Epik at pH 7.4 ± 2.0. The hydrogen-bonding network was subsequently optimized using PROPKA at pH 7.4, including optimization of the orientations of retained water molecules. Finally, the prepared protein was subjected to restrained minimization using the OPLS4 force field until a maximum heavy-atom RMSD of 0.30 Å relative to the crystallographic coordinates was reached.

### 4.2. Ligand Library Preparation and Refinement for High-Throughput Screening

Approximately 215,000 potential antiviral compounds were systematically sourced from multiple databases, including DrugBank, Thermo Fisher Scientific Inc. (Waltham, MA, USA) (https://www.thermofisher.com/bd/en/home/chemicals/applications/drug-discovery/high-throughput-screening.html/; accessed on 4 December 2024), and PubChem (based on the existing literature on NiV) [51]. These compounds included bioactive molecules, metabolites, nutraceuticals, and both approved and experimental drugs. The compounds were initially processed using OpenBabel to convert them into SDF format, and any duplicate entries were removed during this step.

The LigPrep module of the Schrödinger suite (Schrödinger Release 2023-2) was used to optimize and standardize the ligand structures (Schrödinger, LLC, New York, NY, USA). The Epik tool from the LigPrep module was used to generate the appropriate ionization states at physiological pH [52]. Moreover, the structures were subsequently optimized using the OPLS4 force field [53]. The prepared ligand structures were evaluated using QikProp and subjected to Lipinski-based physicochemical filtering using the following criteria: molecular weight ≤ 500 Da, QPlogP(o/w) ≤ 5, hydrogen-bond donors ≤ 5, and hydrogen-bond acceptors ≤ 10. In addition, structures containing predicted reactive groups were excluded using the criterion Reactive_groups = 0. Because LigPrep module of the Schrödinger suite (Schrödinger Release 2023-2) generated multiple ionization, tautomeric, and permitted stereochemical states from individual parent compounds, the number of prepared structures was greater than the number of initial compounds. Following ligand preparation and physicochemical/reactivity filtering, 302,577 prepared ligand structures were retained for high-throughput virtual screening.

### 4.3. Defining the Receptor Grid for Docking and Binding-Site Identification

**Figure 8:**
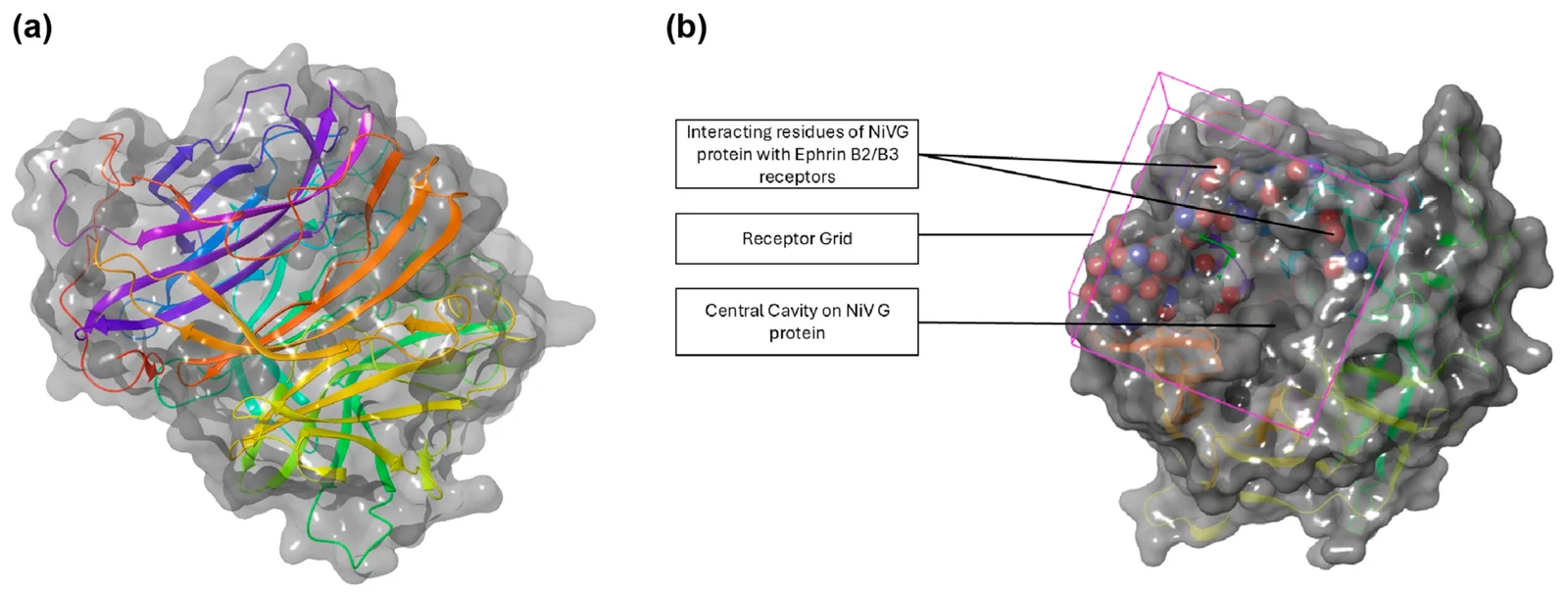
(**a**) Crystal structure of the attachment G glycoprotein of NiV (PDB ID—3D11). (**b**) 3D structure of the NiV G attachment glycoprotein with receptor grid for docking studies. The highlighted regions show the amino acid residues on NiV G (in a space-filling model) that interact with the ephrin receptor, while the receptor grid (depicted as a purple box) defines the binding site for molecular docking analysis.

We defined a grid box on the target protein for docking, because it is essential to accurately define a grid box over the functional region of the target protein for ensuring a focused docking. In this study, the Glide grid generation panel from the Schrödinger suite was used to create the receptor grid [54,55]. This grid covers the area containing key amino acid residues on the NiV G protein that interact with ephrin receptors on the host cells, as shown in Figure 8b [38,56]. The grid was centered at x = 30.0, y = 5.0, and z = 100.0 Å in the coordinate frame of PDB entry 3D11. An inner box of 10 × 10 × 10 Å and an enclosing box of 20 × 20 × 20 Å were used. The binding region included the residues listed in Table 3 [57]. No hydrogen-bond, positional, or metal-coordination constraints were applied, and no rotatable receptor hydroxyl groups were defined. For ligand docking, van der Waals radii were scaled by 0.80 for ligand atoms with partial charges below 0.15.

### 4.4. Molecular Docking Workflow for Identifying Potential Inhibitors

Structure-based screening is a valuable method for identifying potential drug candidates by evaluating how well molecules interact with the target protein’s active site [58]. This approach involves scanning large ligand databases to identify compounds that fit the protein’s binding site [59]. Virtual screening was performed using the GLIDE module in the Schrödinger suite, which was conducted in three phases. First, high-throughput virtual screening (HTVS) was applied to the ligand library, identifying the top 30% of molecules based on their docking scores [54,55]. Virtual screening was performed using the GLIDE module in the Schrödinger suite (Schrödinger, LLC, New York, NY, USA), which was conducted in three phases. First, all 302,577 prepared ligand structures that passed the ligand-preparation and filtering steps were subjected to high-throughput virtual screening (HTVS). The top 30% based on docking performance were retained, resulting in 90,773 structures. These structures were subsequently subjected to standard precision (SP) docking, from which the top 20% were retained, yielding 18,154 structures. Extra precision (XP) docking was then performed on the SP-selected structures, and the top 10% were retained, resulting in 1815 structures.

All 1815 XP-selected structures were subsequently subjected to MM-GBSA binding-energy estimation. Following evaluation of the docking scores, estimated MM-GBSA values, and two-dimensional protein–ligand interaction profiles at the target site, four compounds were selected for further molecular dynamics simulations and experimental evaluation.

### 4.5. Estimation of Ligand Binding Free Energy via the MM-GBSA Approach

Compounds from the XP docking results were selected for MM-GBSA (Molecular Mechanics Generalized Born Surface Area) estimation to assess their binding affinity. We used the Prime MM-GBSA method from the Schrödinger suite (Schrödinger Release 2023-2) to calculate the binding free energies of the ligand–protein interactions. This method combines molecular mechanics with solvation energies, utilizing the OPLS-2005 force field and the VSGB solvation model [53,60]. The use of OPLS-2005 at this stage was consistent with the force field used for receptor-grid generation, Glide docking, and the subsequent molecular dynamics simulations. The docked poses generated from the Extra Precision (XP) docking in the GLIDE module were processed for estimation of binding free energy via the MM-GBSA approach. The binding free energy (ΔG bind) was computed using the following formula:

ΔG bind = E Complex_(minimized)_ − E Ligand_(minimized)_ − E Receptor_(minimized)_

This equation provides a quantitative measure of the binding affinity [61]. Reported ΔG bind values represent single-point Prime MM-GBSA calculations performed on the top-ranked Glide XP docking pose for each compound. As this is a deterministic calculation on a single energy-minimized structure rather than an ensemble or replicate measurement, no statistical error term is associated with these values; this is consistent with standard practice for MM-GBSA-based virtual screening rankings.

### 4.6. Pharmacokinetic and Toxicity Prediction for Potential Inhibitors

Drug-like molecules typically exhibit favorable ADMET (absorption, distribution, metabolism, excretion, and toxicity) properties. To assess the drug-likeness and pharmacokinetic profiles of potential NiV entry inhibitors, Schrodinger’s QikProp module was used (Schrödinger Release Notes—Release 2023-2). This tool compares the properties of a given molecule against the profiles of 95% of known drugs, helping to predict how well the compounds might behave in a biological system [62]. To evaluate the reliability of the four shortlisted docking hits and screen for potential assay-interference liabilities, all candidate structures were assessed using RDKit’s (v2026.03) built-in PAINS and Brenk structural alert filters via the FilterCatalog module. Additionally, they were evaluated against REOS-style property filters (molecular weight 200–500, calculated logP −5 to 5, hydrogen-bond donors 0–5, hydrogen-bond acceptors 0–10, rotatable bonds 0–8, formal charge −2 to +2, heavy-atom count 15–50).

### 4.7. Molecular Dynamics Simulations for Evaluating Protein–Ligand Complex Stability

Molecular dynamics (MD) simulation is an essential tool for evaluating the stability of protein–ligand complexes [63]. In this study, we performed MD simulations on the four most promising protein–ligand complexes for 200 ns using Desmond (Schrödinger Release 2023-2) and the OPLS 2005 force field [64]. The system was set up in a cubic box with a 15 Å buffer around the protein–ligand (P-L) complex. The TIP3P water model was employed for solvation [65], and physiological conditions were mimicked by adding 0.15 M NaCl. The simulation was conducted at a constant temperature of 300 K and pressure of 1 bar. Ions were added to ensure the system’s electrical neutrality. The system was relaxed using Desmond’s default multi-stage protocol: Brownian dynamics at 10 K in the NVT ensemble for 100 ps with harmonic restraints on solute heavy atoms, followed by restrained NVT at 10 K (12 ps), restrained NPT at 10 K (12 ps), restrained NPT at 300 K (12 ps), and finally unrestrained NPT at 300 K (24 ps). Production dynamics were run for 200 ns in the NPT ensemble at 300.0 K and 1.01325 bar. Temperature and pressure were maintained by a Nosé–Hoover chain thermostat (relaxation time 1.0 ps) and a Martyna–Tobias–Klein barostat (relaxation time 2.0 ps, isotropic coupling). A RESPA integrator was used with a 2.0 fs timestep for bonded and short-range non-bonded forces and a 6.0 fs timestep for long-range electrostatics. Coordinates were saved every 200 ps, giving approximately 1000 frames for trajectory analysis.

To assess the stability of the protein–ligand complexes, we analyzed a single simulation trajectory for each protein–ligand complex using several metrics: root mean square fluctuation (RMSF), protein–ligand contact mapping, and protein–ligand contact timeline. Ligand RMSD was calculated for the ligand atoms following least-squares superposition (fitting) of the protein backbone Cα atoms of each trajectory frame onto the initial reference structure, so that ligand displacement was measured relative to the protein frame of reference rather than to the ligand’s own initial position. Protein RMSD was calculated analogously using the backbone Cα atoms of the protein throughout the trajectory. To characterize the dominant protein–ligand interaction states within the stable region of the trajectory (100–200 ns), per-frame contact fingerprints were constructed from the hydrogen-bond, hydrophobic, ionic, metal/ion-bridged, π-cation, π-π, and water-bridged interaction records exported from the Simulation Interaction Diagram. Frames were clustered on these interaction fingerprints using k-means (k = 3, scikit-learn v1.9.0), and the dominant cluster’s characteristic contacts were reported as the representative interaction state for this period. Since hydrogen bonds play a crucial role in stable and accurate binding [66,67], we also evaluated the proportion of hydrogen-bond contacts over time to understand the stability and occupancy of potential NiV entry inhibitors within the receptor-binding cavity.

Based on the results from molecular docking and MD simulation studies, one compound (quercetin) was selected for cell-based experimental validation.

### 4.8. Cell Line

Vero cells (ATCC CCL-81) were obtained from the American Type Culture Collection (ATCC, Manassas, VA, USA). The cells were cultured using Minimum Essential Medium (MEM; Sigma-Aldrich, St. Louis, MO, USA) supplemented with 10% Fetal Bovine Serum (FBS; Gibco, Thermo Fisher Scientific, Waltham, MA, USA), 1% Penicillin/Streptomycin (Sigma), 1% HEPES buffer (Sigma), and 1% L-glutamine (200 mM, Sigma) at 37 °C in a humidified 5% CO incubator.

### 4.9. Virus Culture

A recombinant Cedar virus (rCedV) encoding the NiV F and G glycoproteins and Green Fluorescent Protein (GFP) (rCedV-NiV-B-GFP) was used to assess the virus inhibitory activity of compounds [26]. The virus was propagated in Vero cells following the original protocol under biosafety level-2 (BSL-2) conditions [68].

In brief, a T-75 culture flask was seeded with Vero cells and incubated until the cells reached about 80% confluency. Then, the culture medium was replaced with MEM supplemented with 2% FBS (MEM-2). The cells were infected with rCedV-NiV-B-GFP at a multiplicity of infection (MOI) of 0.001. The infected cells were incubated until apparent cytopathic effects (CPE), characterized by syncytia, were observed, which took 3–4 days. Culture supernatant was collected and centrifuged at 948 rcf to remove cellular debris. The clear supernatant was then filtered-sterilized, aliquoted, and stored at −80 °C.

### 4.10. Plaque Assay

Viral titer of the rCedV-NiV-B-GFP was measured by plaque assay using Vero cells. Cells were seeded in a 6-well plate so that each well contained 5.0 × 10^5^ cells and were incubated at 37 °C with 5% CO_2_ overnight to achieve 80–90% confluency. Tenfold serial dilutions (10^−1^ to 10^−6^) of virus stock were prepared in MEM-2. The culture medium was removed from the 6-well plate, and the wells were inoculated with 0.5 mL diluted virus solutions. After 1 h of incubation at 37 °C, the virus solutions were removed. 2 mL of overlay medium was added to each well. The overlay medium was prepared by a 1:1 mixture of MEM-5 (MEM supplemented with 5% FBS) and 2% carboxymethylcellulose (CMC). The plate was incubated for 4 days to allow plaque formation.

Following incubation, the overlay medium was removed, and cells were fixed with 10% formaldehyde in 1x PBS for 1 h at room temperature. The fixed cells were stained with 0.5% crystal violet solution in 25% methanol for approximately 30 min. Then, the plate was carefully washed with ultrapure water and air-dried. Visible plaques were counted, and viral titers were calculated using the following formula:

Viral Titer (PFU⁄mL) = Average Number of Plaques × Dilution Factor × 2

Here, the factor of 2 accounts for the 0.5 mL inoculum volume used per well.

### 4.11. Compound Preparation

Quercetin (Sigma-Aldrich, St. Louis, MO, USA, Cat. No. Q4951) was obtained as a powder and dissolved in 100% DMSO to prepare a 20 mg/mL solution, which was subsequently diluted 1:1 with MEM to obtain a 10 mg/mL stock containing 50% (*v*/*v*) DMSO. Quercetin was further diluted in assay medium to the required test concentrations. At the highest final quercetin concentration used in the neutralization assays (30.22 μg/mL), the final DMSO concentration was approximately 0.15% (*v*/*v*). Because quercetin was prepared by two-fold serial dilution, the DMSO concentration decreased proportionally with each subsequent dilution. Ascorbic acid (Sigma-Aldrich, Cat. No. A4403) was dissolved in ultrapure water to prepare a 10 mg/mL stock solution. Both stock solutions were filtered and sterilized by passing through a 0.22 µm filter.

### 4.12. Cytotoxicity Assay

The cytotoxicity of the drug-like compounds was evaluated in Vero cells using the MTS colorimetric assay. Vero cells were seeded into a 96-well plate at a density of 2.0 × 10^4^ cells/well in 100 µL of culture media. The plate was incubated at 37 °C in a CO_2_ incubator for 16–24 h to allow cell confluency to reach 80–90%. Then, the culture medium was replaced with 100 µL of drug-containing assay medium (MEM supplemented with 5% FBS), and the negative control wells contained only assay medium with cells. The plate was then incubated for 48 h at 37 °C and 5% CO_2_. Following the 48 h treatment period, the drug-containing medium was completely removed from each well and replaced with fresh, compound-free assay medium to minimize potential direct chemical interaction between quercetin and/or ascorbic acid and the MTS reagent. Then 20 µL of MTS was added to each well and incubated for 6 h at 37 °C. Then, absorbance was measured at 450 nm wavelength using a multiplate reader. Cell viability was reported after subtraction of the cell-free signal at different concentrations.

### 4.13. Fluorescence Reduction Neutralization Test

The antiviral properties of the drug-like compounds were evaluated using the FRNT assay. A 96-well black, clear-bottom plate was seeded with Vero cells in culture medium, using 100 μL of solution and 2.0 × 10^4^ cells per well. The cells were incubated at 37 °C and 5% CO_2_ until 80–90% confluency was reached. The compounds and virus stock solutions were diluted in an assay medium containing MEM supplemented with 5% FBS. The virus was diluted to achieve a final multiplicity of infection (MOI) of 0.05 per well. Equal volumes of drug and virus were mixed, shaken (200 rpm, 3–5 min), and incubated for 2 h at 37 °C, 5% CO_2_. After removing the cell culture medium, 100 μL of the drug–virus mixture was transferred to the Vero-seeded plate. The virus controls contained rCedV-NiV-B-GFP and cells with assay media; the cell-only control wells contained cells with assay medium; and the positive-virus neutralization control contained mAb-7B7 against Nipah G glycoprotein (Absolute Antibody, Cat. No. Ab02864), rCedV-NiV-B-GFP, and cells. The plate was incubated at 37 °C, 5% CO_2_ for 24 h to allow the expression of GFP.

At the end of the incubation, the medium was removed, and cells were fixed using 4% formaldehyde for 20 min. The plate was then washed three times with ultrapure water. After air-drying, the plate was imaged using the CTL ImmunoSpot Analyzer’s GFP channel. The resulting images were analyzed using the CTL ImmunoSpot Analyzer’s dedicated software to count discrete GFP-positive foci in each well automatically.

### 4.14. Statistical Analysis

Each assay was performed in three independent experiments, with three technical replicate wells per condition in each experiment. Percent cell viability was calculated by normalizing absorbance values to cell and media controls in GraphPad Prism 10 (GraphPad Software, Boston, MA, USA). Percent viral neutralization was determined by comparing infection foci counts relative to cell and virus controls. 50% cytotoxic concentration (CC_50_) values, dose–response curves, and half-maximal inhibitory concentration (IC_50_) values were calculated using a four-parameter log-logistic regression model in GraphPad Prism 10. Drug–drug interaction and synergistic effects were evaluated using the SynergyFinder R package (v3.7.4) [47], with dose–response data reshaped via ReshapeData() (impute = TRUE, noise = FALSE, seed = 1, iteration = 10, data_type = “inhibition”) and synergy scores calculated via CalculateSynergy() using the Highest Single Agent (HSA) and Zero Interaction Potency (ZIP) models. For each combination dose, the HSA reference is defined as the greater of the two single-agent inhibition responses at the corresponding individual concentrations, and the HSA synergy score is the difference between the observed combination response and this reference. The overall HSA synergy score reported is the mean across all non-single-agent dose combinations within the 4 × 4 matrix (quercetin and ascorbic acid, each at 0, 25, 50, and 100 µM), based on three technical replicates per combination. Because dose–response data were collected in three technical replicates at each concentration combination, synergy scores and their statistical significance were calculated using the package’s replicate-aware bootstrap procedure. The response matrix was resampled with replacement over 10 bootstrap iterations, the synergy score was recalculated for each resample, and the resulting distribution of the mean synergy score across the combination doses was used to compute a z-statistic and corresponding *p*-value using the normal-tail approximation described by Zelen and Severo [69].

## 5. Conclusions

In summary, this study provides structure-guided identification and experimental evaluation of quercetin as a potential inhibitor of NiV in an rCedV-NiV-B-GFP surrogate neutralization system. Computational analyses suggest that the ephrin-binding region of the NiV-G attachment glycoprotein may serve as a potential molecular target. In addition, ascorbic acid enhanced the inhibitory activity of quercetin, with synergistic effects observed under the tested conditions against rCedV-NiV-B-GFP. This integrated computational and experimental approach provides a robust framework for developing antiviral therapeutics against NiV and related henipaviruses.

## Figures and Tables

**Table 1:** Results of docking and free energy of binding.

Serial No	Compound PubChem ID (CID)	Docking Score	MM-GBSA ΔG Bind (kcal/mol)
1	5280343 (Quercetin)	−8.381	−34.22
2	2815223	−8.241	−43.13
3	104762	−7.872	−3.48
4	25163987	−7.674	−52.15

**Table 2:** Predicted (in silico) Pharmacokinetic and Drug-Likeness Profiles of Ligands.

Criteria *	Quercetin	CID 2815223	CID 104762	CID 25163987	Acceptable Range
QPpolrz	27.523	32.771	20.454	34.961	13.0 to 70.0
QPlogPC16	10.745	10.536	8.880	12.385	4.0 to 18.0
QPlogPoct	18.560	16.430	21.141	21.099	8.0 to 35.0
QPlogPw	14.423	10.055	18.820	13.234	4.0 to 45.0
QPlogPo/w	0.364	2.759	−2.012	2.962	−2 to 6.5
QPlogS	−2.901	−4.677	−1.483	−4.922	−6.5 to 0.5
QPlogHERG	−5.097	−5.286	−3.451	−5.667	Concern below −5
QPPCaco	18.195	394.974	11.067	270.735	<25 = poor, >500 = Great
QPlogBB	−2.416	−1.103	−2.404	−1.194	−3 to 1.2
QPPMDCK	6.510	317.834	3.804	1485.298	<25 = poor, >500 = Great
QPlogKp	−5.546	−3.175	−6.550	−2.992	−8 to −1
QPlogKhsa	−0.344	0.154	−0.878	−0.135	−1.5 to 1.5
HOAP	51.629	89.574	20.895	87.827	<25 = poor
#metab	5	4	6	4	1 to 8
#rtvFG	0	0	0	1	0 to 2
CNS	−2	−2	−2	−2	−2 to +2
Glob	0.8475100	0.8169000	0.8923326	0.8032060	0.75 to 0.95
Rule of Five	0	0	0	0	0 to 1

* QPolrz quantifies polarizability; **QPlogPC16** and **QPlogPoct** assess hydrophobicity via hexadecane/gas and octanol/gas partition coefficients, respectively. **QPlogPw** represents aqueous solubility. **QlogPo/w** and **QPlogS** measure lipophilicity and solubility, respectively. **CIQPlogS** measures confirmation-independent solubility. **QPlogHREG** predicts IC_50_ for HERG K+ channel inhibition, critical for cardiac safety. **QPPCaco** estimates Caco-2 permeability, a surrogate for gastrointestinal absorption, while **QPlogBB** and **QPPMDCK** assess brain–blood and MDCK permeability, indicating CNS and cellular penetration. **QPlogKp** measures skin permeability, **QPlogKhsa** forecasts binding affinity to human serum albumin, and HOA (%) estimates oral absorption. **#metab** indicates metabolic stability, **#rtvFG** counts reactive functional groups, **CNS** predicts CNS activity, and **Globularity** **(glob)** evaluates molecular shape. **Rule of Five** checks adherence to Lipinski’s criteria for oral drug potential (MW < 500, logPo/w < 5, donors < 5, acceptors < 10).

**Table 3:** Targeted amino acid residues for blocking NiV entry into host cells.

Criteria	Position	Type of Amino Acid
Interacting residues of NiVG protein with Ephrin B2/B3 receptors	504	TRP
	505	GLU
	530	GLN
	531	THR
	532	ALA
	533	GLU
	557	ASN
	579 to 590	A flexible loop on the NiVG surface that plays a critical role in NiVG–ephrin complex formation.

## Data Availability

The data supporting the findings of this study are provided within the article and its Supplementary Materials. The accompanying supplementary Excel workbook includes the raw FRNT focus counts, MTS absorbance values, curve-fitting data, complete synergy analysis data, and virtual screening ranking. All supplementary tables can be found in the file named Supplementary Tables. Additional information may be obtained from the corresponding author upon reasonable request.

## Supplementary Materials

The following supporting information can be downloaded at: https://www.mdpi.com/article/10.3390/ijms27188026/s1, All supplementary tables are in a file named Supplementary Tables.
