# Supplementary material for: Discovery of Quercetin as a Potential Entry Inhibitor of Nipah Virus: A Path Toward Antiviral Therapy
Source: Int J Mol Sci. 2026 Sep 9;27(18):8026. doi: 10.3390/ijms27188026 (PMC13606494; doi:10.3390/ijms27188026)
Supplement: Supplementary file 1 [file ijms-27-08026-s001.zip › ijms-4496437-supplementary.pdf]

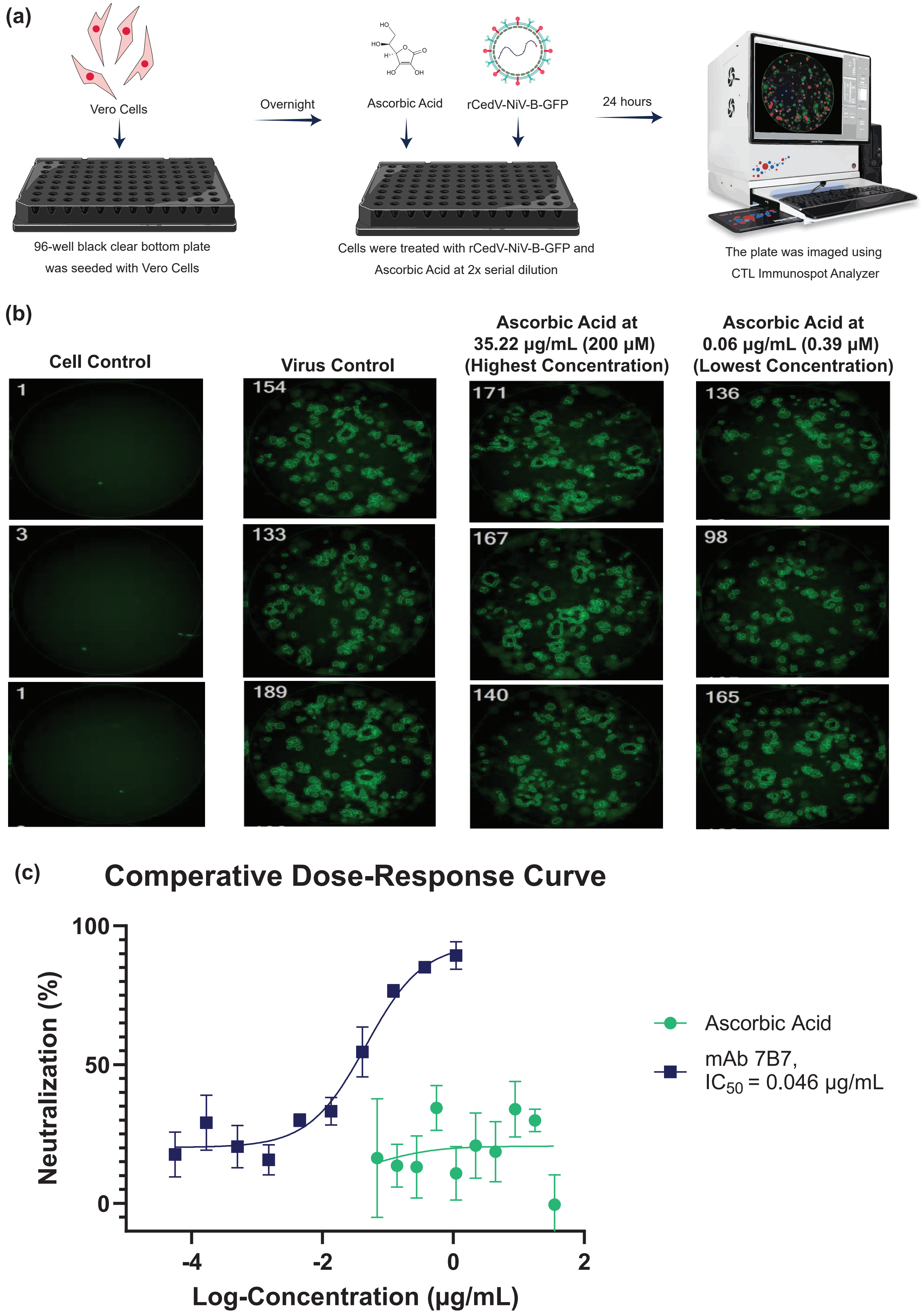
(a)
Vero Cells
96-well black clear bottom plate
was seeded with Vero Cells
Overnight
Ascorbic Acid
rCedV-NiV-B-GFP
Cells were treated with rCedV-NiV-B-GFP and
Ascorbic Acid at 2x serial dilution
24 hours
The plate was imaged using
CTL Immunospot Analyzer
(b)
Cell Control
Virus Control
Ascorbic Acid at
35.22 µg/mL (200 µM)
(Highest Concentration)
Ascorbic Acid at
0.06 µg/mL (0.39 µM)
(Lowest Concentration)
1
3
1
154
133
189
171
167
140
136
98
165
(c)
Comperative Dose-Response Curve
Neutralization (%)
100
50
0
-4
-2
0
2
Log-Concentration (µg/mL)
Ascorbic Acid
mAb 7B7,
IC_{50} = 0.046 µg/mL

Supplementary Figure S1: Antiviral activity of Ascorbic Acid against NiV using rCedV-NiV-B-GFP dependent FRNT. (a) Schematic overview of the FRNT. Vero cells were seeded in a 96-well black clear bottom plate and incubated overnight. Cells were then treated with Ascorbic Acid at 2-fold dilutions (from 200 µM to 0.39 µM) and GFP-expressing rCedV encoding NiV glycoproteins. Following 24-hours of incubation, the plate was fixed, washed, and imaged using the CTL Immunospot Analyzer and quantified GFP-positive foci. (b) Representative fluorescence microscopy images of FRNT wells. Images show GFP-positive foci (green) for the cell control (uninfected), the virus control (rCedV-NiV-B-GFP only), and wells treated with the highest (35.22 µg/mL or, 200 µM) and lowest (0.06 µg/mL or, 0.39 µM) concentrations of Ascorbic Acid. The number in the upper left corner of each image represents the automatically counted number of foci. No significant reduction in foci count in the presence of 35.22 µg/mL Ascorbic Acid compared to the virus control was observed. (c) Dose-dependent neutralization curve for Ascorbic Acid and positive control (mAb 7B7). The neutralization percentage is plotted against the Log concentration (µg/mL) for Ascorbic Acid (green circles) and the positive control mAb 7B7 (blue squares). Data were fitted to a non-linear regression curve to determine the IC_{50}. The calculated IC_{50} for mAb was 0.046 µg/m, however, the Ascorbic Acid didn't show any dose-dependent neutralization effect. Error bars represent the standard deviation of three replicate wells (n=3).

Supplementary Table S1: Raw FRNT focus counts

Concentration (ug/mL)	Quercetin			Quercetin + 25 uM AA			Quercetin + 50 uM AA			Quercetin + 100 uM AA		
30.224	6	12	27	3	2	3	8	2	3	2	2	0
15.112	32	14	37	2	0	5	0	2	0	5	3	3
7.556	65	60	44	19	10	16	2	2	4	7	4	11
3.778	70	80	69	11	31	29	26	32	33	19	9	18
1.889	59	77	105	76	56	48	45	69	28	53	76	58
0.9445	72	119	82	46	47	64	49	67	55	33	51	55
0.47225	137	137	93	107	103	113	90	102	65	77	67	92
0.236125	74	113	91	114	117	115	87	134	102	98	99	97
0.118063	72	92	117	128	111	118	95	90	103	109	82	120
0.059031	136	133	128	108	124	111	78	110	159	74	125	117

Concentration (ug/mL)	mAb 7B7	
1.1	7	8
0.366667	24	14
0.122222	48	29
0.040741	63	77
0.01358	64	60
0.004527	102	107
0.001509	108	113
0.000503	140	104
0.000168	125	116
0.0000559	132	99

Supplementary Table S2: MTS absorbance values (450nm)

Concentration (uM)	Quercetin		
500	1.535	1.628	1.647
250	2.482	2.522	2.461
125	2.278	2.351	2.167
62.5	2.608	2.291	2.137
31.25	2.477	2.312	2.467
15.625	2.665	2.562	2.316
7.8125	2.398	2.429	2.343
3.90625	2.243	2.21	2.241
1.953125	2.695	2.243	2.428
0.9765625	2.204	2.161	2.393
Cell Control	2.322	2.235	2.271

Concentration (uM)	Quercetin + 25 uM AA		
500	1.147	1.25	1.243
250	1.564	1.576	1.594
125	1.5	1.53	1.531
62.5	1.559	1.569	1.564
31.25	1.666	1.728	1.736
15.625	1.738	1.621	1.685
7.8125	1.833	1.813	1.841
3.90625	1.768	1.669	1.709
Cell Control	1.665	1.605	1.619

Supplementary Table S3: Curve-fitting data

**Curve-fitting data:** FRNT					
log(inhibitor) vs. response (three parameters)	Quercetin	Quercetin + 25 uM AA	Quercetin + 50 uM AA	Quercetin + 100 uM AA	mAb 7B7
Best-fit values					
Bottom	106.6	105.1	105.1	104.5	90.76
Top	13.72	0.3312	9.04	12.94	8.982
LogIC50	0.8611	0.1231	0.1008	0.08822	-1.557
IC50	7.263	1.328	1.261	1.225	0.02771
Span	-92.83	-104.7	-96.08	-91.57	-81.78
95% CI (profile likelihood)					
Bottom	79.49 to 174.1	96.73 to 114.3	93.70 to 118.5	94.65 to 115.9	77.95 to 106.5
Top	2.873 to 23.31	-8.892 to 8.736	-4.928 to 20.84	0.7310 to 23.29	-0.1118 to 17.51
LogIC50	0.3625 to 1.452	-0.06288 to 0.3118	-0.1975 to 0.3978	-0.1876 to 0.3615	-1.968 to -1.134
IC50	2.304 to 28.30	0.8652 to 2.050	0.6346 to 2.499	0.6492 to 2.299	0.01076 to 0.07346
Goodness of Fit					
Degrees of Freedom	27	27	27	27	17
R squared	0.7606	0.9405	0.8724	0.895	0.9098
Sum of Squares	6315	2378	4627	3371	1954
Sy.x	15.29	9.385	13.09	11.17	10.72

**Curve-fitting data:** Cytotoxicity Assay		
log(inhibitor) vs. normalized response -- Variable slope	Quercetin	Quercetin + 25 uM AA
Best-fit values		
LogIC50	2.722	2.917
HillSlope	-4.611	-1.237
IC50	527	825.9
95% CI (profile likelihood)		
LogIC50	2.676 to 2.944	2.771 to 3.181
HillSlope	??? to -1.177	-2.175 to -0.8015
IC50	473.8 to 879.8	590.6 to 1515
Goodness of Fit		
Degrees of Freedom	28	22
R squared	0.425	0.7885
Sum of Squares	2898	956.3
Sy.x	10.17	6.593

Supplementary Table S4: Synergistic effect analysis data.						
**Compound_1**	**Compound_2**	**Quercetin_Concentration**	**Ascorbic_Acid_Concentration**	**Response**	**Concentration_Unit**	**Replicate**
Quercetin	Ascorbic_Acid	100	100	98.70542636	µM	1
Quercetin	Ascorbic_Acid	50	100	96.37984496	µM	1
Quercetin	Ascorbic_Acid	25	100	94.82945736	µM	1
Quercetin	Ascorbic_Acid	100	50	94.05426357	µM	1
Quercetin	Ascorbic_Acid	50	50	100.255814	µM	1
Quercetin	Ascorbic_Acid	25	50	98.70542636	µM	1
Quercetin	Ascorbic_Acid	100	25	97.93023256	µM	1
Quercetin	Ascorbic_Acid	50	25	98.70542636	µM	1
Quercetin	Ascorbic_Acid	25	25	85.52713178	µM	1
Quercetin	Ascorbic_Acid	100	100	98.70542636	µM	2
Quercetin	Ascorbic_Acid	50	100	97.93023256	µM	2
Quercetin	Ascorbic_Acid	25	100	97.15503876	µM	2
Quercetin	Ascorbic_Acid	100	50	98.70542636	µM	2
Quercetin	Ascorbic_Acid	50	50	98.70542636	µM	2
Quercetin	Ascorbic_Acid	25	50	98.70542636	µM	2
Quercetin	Ascorbic_Acid	100	25	98.70542636	µM	2
Quercetin	Ascorbic_Acid	50	25	100.255814	µM	2
Quercetin	Ascorbic_Acid	25	25	92.50387597	µM	2
Quercetin	Ascorbic_Acid	100	100	100.255814	µM	3
Quercetin	Ascorbic_Acid	50	100	97.93023256	µM	3
Quercetin	Ascorbic_Acid	25	100	91.72868217	µM	3
Quercetin	Ascorbic_Acid	100	50	97.93023256	µM	3
Quercetin	Ascorbic_Acid	50	50	100.255814	µM	3
Quercetin	Ascorbic_Acid	25	50	97.15503876	µM	3
Quercetin	Ascorbic_Acid	100	25	97.93023256	µM	3
Quercetin	Ascorbic_Acid	50	25	96.37984496	µM	3
Quercetin	Ascorbic_Acid	25	25	87.85271318	µM	3
Quercetin	Ascorbic_Acid	0	100	25.26751592	µM	1
Quercetin	Ascorbic_Acid	0	50	37.36942675	µM	1
Quercetin	Ascorbic_Acid	0	25	7.433121019	µM	1
Quercetin	Ascorbic_Acid	0	100	32.27388535	µM	2
Quercetin	Ascorbic_Acid	0	50	41.82802548	µM	2
Quercetin	Ascorbic_Acid	0	25	29.08917197	µM	2
Quercetin	Ascorbic_Acid	0	100	32.27388535	µM	3
Quercetin	Ascorbic_Acid	0	50	22.71974522	µM	3
Quercetin	Ascorbic_Acid	0	25	19.53503185	µM	3
Quercetin	Ascorbic_Acid	100	0	95.60465116	µM	1
Quercetin	Ascorbic_Acid	50	0	75.4496124	µM	1
Quercetin	Ascorbic_Acid	25	0	49.86821705	µM	1
Quercetin	Ascorbic_Acid	100	0	90.95348837	µM	2
Quercetin	Ascorbic_Acid	50	0	89.40310078	µM	2
Quercetin	Ascorbic_Acid	25	0	53.74418605	µM	2
Quercetin	Ascorbic_Acid	100	0	79.3255814	µM	3
Quercetin	Ascorbic_Acid	50	0	71.57364341	µM	3
Quercetin	Ascorbic_Acid	25	0	66.14728682	µM	3
Quercetin	Ascorbic_Acid	0	0	1.80620155	µM	1
Quercetin	Ascorbic_Acid	0	0	3.356589147	µM	2
Quercetin	Ascorbic_Acid	0	0	-5.170542636	µM	3

Supplementary Table S5: Virtual screening ranking

Rank	PubChem CID	Compound	Molecular formula	MW (g/mol)	Glide XP docking score	Prime MM-GBSA ΔGbind (kcal/mol)
1	5280343	Quercetin	C15H10O7	302.23	-8.381	-34.22
2	2815223	[4-(3,4-Dihydroxyphenyl)-1,3-thiazol-2-yl]methyl pivalate	C15H17NO4S	307.4	-8.241	-43.13
3	104762	Mizoribine	C9H13N3O6	259.22	-7.872	-3.48
4	44187362	Pimasertib	C15H15FIN3O3	431.2	-7.766	-45.24
5	667639	Piceatannol	C14H12O4	244.24	-7.677	-38.76
6	25163987	2-[(2-chloro-4-iodophenyl)amino]-N-{[(2R)-2,3-dihydroxypropyl]oxy}-3,4-difluorobenzamide	C16H14ClF2IN2O4	498.65	-7.674	-52.15
7	46937145	(4S)-4-[4-(2,5-dioxopyrrolidin-1-yl)anilino]-4-hydroxybutanoic acid	C14H16N2O5	292.29	-7.425	-4.07
8	9562794	(E)-3,4-Dihydroxy-N'-((2-methoxynaphthalen-1-yl)methylene)benzohydrazide	C19H16N2O4	336.3	-7.332	-45.55
9	6449876	Terbogrel	C23H27N5O2	405.5	-7.324	-24.2
10	100802	Cytochlor	C9H12ClN3O4	261.66	-7.185	-34.98
